# Genomic and geographical structure of human cytomegalovirus

**DOI:** 10.1073/pnas.2221797120

**Published:** 2023-07-17

**Authors:** Oscar J. Charles, Cristina Venturini, Soren Gantt, Claire Atkinson, Paul Griffiths, Richard A. Goldstein, Judith Breuer

**Affiliations:** ^a^Department of Infection, Immunity and Inflammation, University College London, Great Ormond Street Institute of Child Health, London WC1N 1EH, United Kingdom; ^b^Research Centre of the Sainte-Justine University Hospital and Department of Microbiology, Infectious Diseases and Immunology, University of Montréal, Montréal, Quebec H3T 1C5, Canada; ^c^Division of Infection and Immunity, Institute for Immunity and Transplantation, University College London, London NW3 2PP, United Kingdom; ^d^Division of Infection and Immunity, University College London, London WC1E 6BT, United Kingdom; ^e^Great Ormond Street Hospital for Children National Health Service Foundation Trust, London WC1N 1LE, United Kingdom

**Keywords:** human cytomegalovirus, genotyping, hypervariability, hidden Markov models, genomics

## Abstract

Human cytomegalovirus (CMV) has infected humans since the origin of our species and currently infects most of the world’s population. Variability between CMV genomes is the highest of any human herpesvirus, yet large portions of the genome are conserved. Here, we show that the genome encodes 74 regions of relatively high variability each with 2 to 8 alleles. We then identified two patterns in the CMV genome. Conserved parts of the genome and a minority (32) of variable regions show geographic population structure with evidence for African or European clustering, although hybrid strains are present. We find no evidence that geographic segregation has been driven by host immune pressure affecting known antigenic sites. Forty-two variable regions show no geographical structure, with similar allele distributions across different continental populations. These “nongeographical” regions are significantly enriched for genes encoding immunomodulatory functions suggesting a core functional importance. We hypothesize that at least two CMV founder populations account for the geographical differences that are largely seen in the conserved portions of the genome, although the timing of separation and direction of spread between the two are not clear. In contrast, the similar allele frequencies among 42 variable regions of the genome, irrespective of geographical origin, are indicative of a second evolutionary process, namely balancing selection that may preserve properties critical to CMV biological function. Given that genetic differences between CMVs are postulated to alter immunogenicity and potentially function, understanding these two evolutionary processes could contribute important information for the development of globally effective vaccines and the identification of novel drug targets.

## Significance

CMV genome diversity is higher than other human herpesvirus, and recombination is pervasive. Here, using hidden Markov modeling, we describe 74 multiallelic regions, with the remaining 86% of the genome showing lower variability, albeit with single-nucleotide polymorphisms. We demonstrate that CMV diversity is influenced by two distinct evolutionary forces. A founder effect results in geographical segregation affecting the regions of low variability and 32 variable regions. In contrast, the 42 remaining regions, which are enriched for immunomodulatory functions, show so-called balancing selection, resulting in maintenance of equal allele frequencies irrespective of geography. These insights into CMV evolution are likely to provide insights into virus biology and inform the development of drugs and global vaccines.

Human cytomegalovirus (CMV; species Human betaherpesvirus 5) is a member of the *Betaherpesvirinae* that infects circa 66 to 90% of adults in any given country (1). Like all human herpesviruses, CMV is a linear double-stranded DNA virus that causes lifelong latent infection by establishing latency in long-lived cell populations and periodically reactivates, resulting in lytic viral replication (2, 3). CMV causes significant burden of disease in those with compromised immune systems (4) and is also the most common infectious cause of congenital disability worldwide (5). Because of this, developing a vaccine is a high public health priority (6).

At approximately 236 kb, CMV has the largest genome of all human herpesviruses (7) and the highest level of genetic diversity of all the known human herpesviruses (8, 9). The virus is known to readily undergo recombination (10, 11), and coinfection is frequently observed, especially in individuals with weakened immune systems (12). Most of the observed CMV diversity occurs in discrete hypervariable regions where sequences cluster into genotypes also known as alleles (13, 14). In some hypervariable genes, e.g., UL55 (glycoprotein B, gB), alleles have been defined (15) and used alone or in combination with other multiallelic regions to genotype CMV and identify mixed infections (16, 17). However, attempts to correlate individual alleles with transmission and pathogenesis have so far been unclear or contradictory (14, 18–24).

Despite the efforts to define CMV diversity, our understanding of the evolutionary history that led to its present variation and whether this variability follows a global pattern is limited. CMV, like other herpesviruses, shows remarkable species-specificity, which results from long-term coevolution and adaptation to the host (25, 26). In contrast, unlike other herpesviruses, human CMV whole genomes from clinical samples show little evidence of geographical or other population structures, with the exception of two Asian genomes that have been shown to cluster phylogenetically (10, 11). This, together with the absence of data from ancient CMV genomes, makes for uncertainty as to when and how current CMV diversity evolved.

To better understand CMV genomic structure and how it has evolved, we employed hidden Markov model (HMM) clustering to delineate the hypervariable and conserved regions across a global and diverse dataset of published and unpublished CMV genomes, which together represent unrelated clinical and low-passage strains (27–29). HMM is able to determine the number of sequence clusters (i.e., alleles) that best explain the diversity across CMV genomes and to identify regions where multiple alleles are present. Using the outputs of the model, we describe precise coordinates for regions of multiallelic variability. We also show that CMV genomes do display geographic population structure and that this is particularly clear within the relatively conserved monoallelic regions of the genomes. We highlight examples of where our approach can help to provide insights for further research into questions of viral pathogenesis and ancient evolution.

## Results

### CMV Diversity and Population Structure Is Determined by 74 Discrete Multiallelic Regions.

We compiled a set of 259 CMV whole genome sequences which had been collected worldwide (*SI Appendix*, Fig. S1, full details are shown in Dataset S1). A total of 233 sequences were retrieved from GenBank (30) with available metadata for 106. Of these 106, 35 were from patients who were immunocompromised through HIV, organ, or bone marrow transplant and 71 were from immune-competent individuals (of whom 60 were congenitally infected babies). Short-read data were available for 17 samples, allowing us to check for mixed infections. Nine samples contained only a single CMV strain, of which five were from HIV-positive and CMV-positive mothers and four were immunocompetent children (primary infection or sibling). Eight samples showed evidence of multiple strains for which we reconstructed the haplotypes (17 sequences in total) with validated methods (8, 31). All eight samples came from HIV-positive mothers with CMV infection (8, 32).

Most genomes were derived from viruses obtained in Europe and the Middle East (n = 216, including Belgium, Czech Republic, France, Germany, Greece, Italy, Netherlands, United Kingdom, and Israel). As Israeli genomes appeared as European in analyses, and the migratory history of the country is complex, we have labeled Israeli sampled genomes as European for simplicity. Thirty sequences (including the reconstructed haplotypes) were from samples collected in Africa (Zambia, Kenya, and Uganda) (17) (32–34). The remaining sequences were collected in other parts of the world: 11 from the United States (America), two from Asia (China and South Korea), and 1 from Australia (Oceania).

**Fig. 1.**
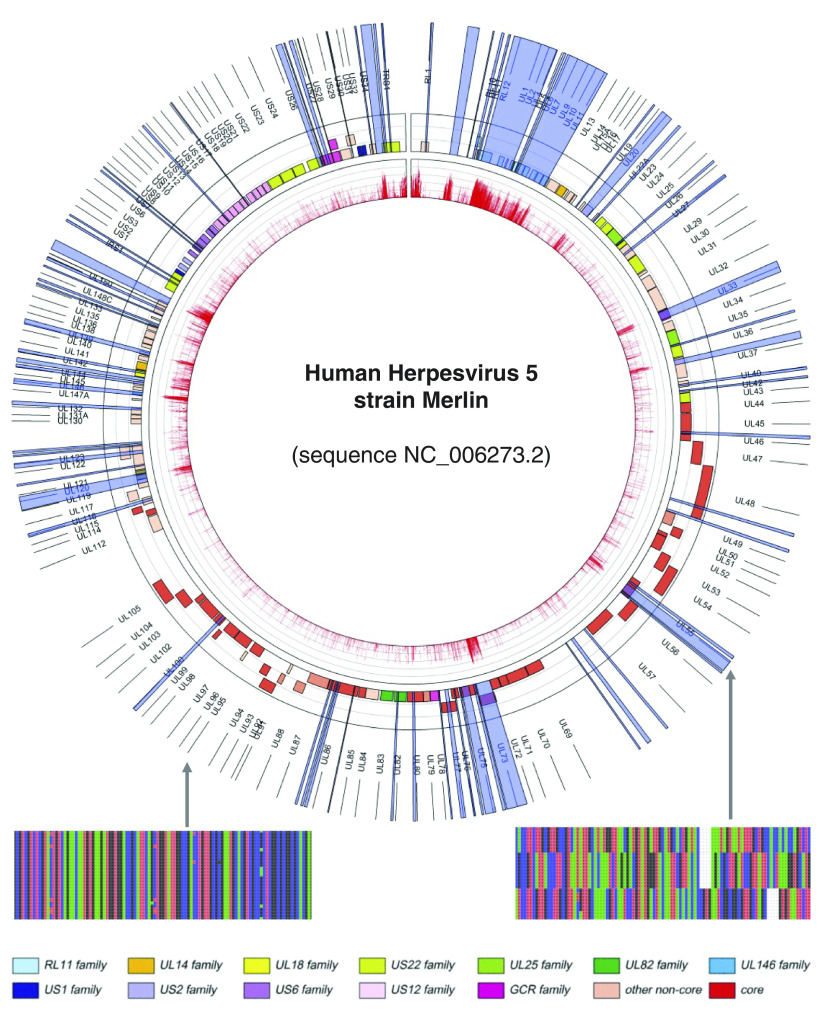
Circular genome map showing nucleotide diversity and multiallelic regions. Tracks numbered from in to out: (track 1, red) Barplot of nucleotide diversity (calculated as heterozygosity) is shown as bars of heterozygosity (red) along a 50-bp moving average; (track 2) open reading frames in the CMV genome are colored by gene family as defined by the bottom legend and in ref. 26; (track 3, blue) Multiallelic regions as defined using HMMcluster are highlighted in translucent blue; (track 4) ORF names. We also show a representative multisequence alignment for conserved (*Left*) and multiallelic (*Right*) regions.

To characterize CMV genomic diversity, we first aligned the genomes and then calculated heterozygosity along a sliding window of 50 base pairs (bp) (track1 in red, Fig. 1). This revealed that while most of the genome is highly conserved, there are regions of significant nucleotide diversity, some of which have previously been described (35, 36). Aligning CMV genomes in these hypervariable regions is challenging and leads to numerous gaps in alignments that are often ignored in phylogenetic and genetic distance calculations. To deal with the complexity of CMV genomes alignment in these regions, we developed a sequence clustering method based on the hidden Markov model called “HMMcluster”. This approach is unaffected by gaps, and it groups together sequences based on the statistical likelihood that they came from the same underlying source. The genome-wide implementation has two steps. In the first, a fixed window is defined (in this case 200 bp, chosen as it returned regions no smaller than 20 bp), and the model calculates the minimum number of sequence clusters (alleles) that best explain the diversity within the window (37). The second step concatenates contiguous windows of sequence variation, and it refines the coordinates for a “variable region”. Variable regions with multiple alleles are defined as “multiallelic”.

From this, we identified 74 discrete variable regions for which the model provided statistical support for the presence of more than one allele (track 3 in blue in Fig. 1 and *SI Appendix*, Tables S1 and S2). These multiallelic regions range in size from 26 to 4,760 nucleotides, encompassing 14% of the genome (Fig. 1 and *SI Appendix*, Table S1). The remaining 86% of the genome was found to be highly conserved with no statistical support for multiple alleles. While multiallelic regions generally occur in regions of high nucleotide diversity, some, generally smaller in size and with fewer segregating single-nucleotide polymorphisms (SNPs), were found in regions of otherwise lower nucleotide diversity. The 74 multiallelic regions were not constrained to known coding regions and encompass the previously documented 12 hypervariable genes (7). Fifty-two of the regions were each contained entirely within a single gene, 17 crossed gene boundaries by either spanning the gap between two genes or extending beyond a gene terminus into noncoding regions, and five regions were entirely in noncoding or otherwise unassigned portions of the genome.

**Fig. 2.**
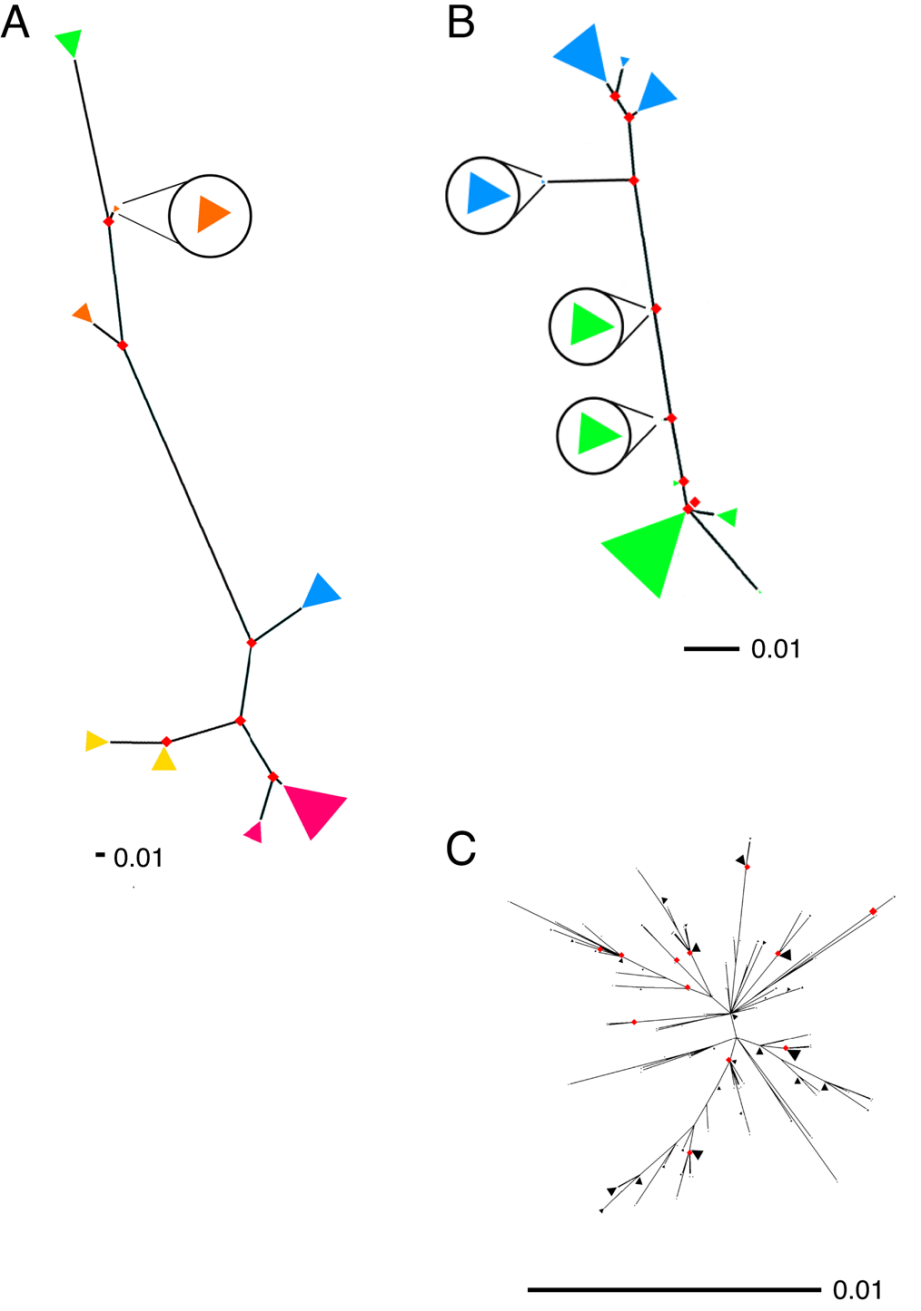
Unrooted maximum likelihood phylogenetic trees of representative multiallelic and conserved regions. Tips were grouped if they were within 5% of the maximum taxa distance and are shown as triangles, where size indicates the number of grouped sequences and color represents a different allele from HMMcluster. Small, hard-to-see fans have been blown up and are represented by fans within circles. Nodes with bootstrap support >90% are shown as red diamonds. Note: Scale bars differ for each figure. (*A*) Multiallelic region 2 (RL5A RL6) (five alleles). (*B*) Multiallelic region 30 (UL75) (two alleles). (*C*) Example conserved region (UL105) (one allele) of comparable alignment length. Variability of *C* is much less than *A* and *B* with no support for HMM-derived clusters. (*D*) *A* and *C* when drawn to the scale of *B*; the example conserved region tree becomes difficult to see at this representation, reflecting the relatively minor variation it encodes. Sequences with greater than 15% ambiguous bases were removed before phylogenetic reconstruction.

To characterize the evolutionary relationships between the alleles within each multiallelic region, we constructed maximum likelihood phylogenetic trees for each of the multiallelic regions, excluding 10 with evidence of recombination breakpoints (Table 1 and *SI Appendix*, Fig. S2). For the remaining 64/74, we observed that multiallelic regions consisted of well-separated clades representing HMMcluster-supported alleles, whereas reconstructed phylogenies of similarly sized conserved regions tended to show poorly resolved clades with low bootstrap support for key central nodes (Fig. 2). Genetic variations in multiallelic regions were as much as an order of magnitude greater than those in comparably sized conserved genome portions (Fig. 2). Well-separated clades with restricted recombination have been identified previously in CMV hypervariable regions and are thought to represent an inability of homologous strands to anneal, supporting the development of population structure within those loci (11).

**Table 1.** Recombination in multiallelic regions

Region	Genes	Number of breakpoints	NC_006273.2 coordinates of breakpoints (bp)
2	RL5A; RL6	2	5,907, 6,290
6	RL11; RL12; RL13; UL1; UL2; UL4	2	12,606, 13,305
8	UL10; UL11; UL6; UL7; UL8; UL9	6	15,751, 15,860, 16,390, 17,553, 18,419, 18,672
10	UL20; UL21A	2	26,468, 26,732
28	UL73; UL74	2	107,640, 107,848
42	UL116	1	166,458
43	UL119; UL120; UL121	6	169,045, 169,283, 169,475, 169,525, 169,782, 170,046
50	UL144	2	182,464, 182,551
51	UL150A	1	183,170
72	TRS1	4	231,683, 231,783, 232,227, 232,415

Each multiallelic region was assessed for evidence of recombination. First, each region was examined using a set of seven recombination methods implemented in RDP5. We then visually investigated the phylogenies either side of predicted breakpoints of those multiallelic regions with evidence of recombination (a region was considered to have evidence of recombination if at least five methods in RDP5 were significant). We removed breakpoints that could be explained by subclade structure. We underline and highlight in bold those regions where most sequences in the multiple-sequence alignment showed the recombination breakpoints.

### European and African Whole Genomes Display Geographical Population Structure.

**Fig. 3.**
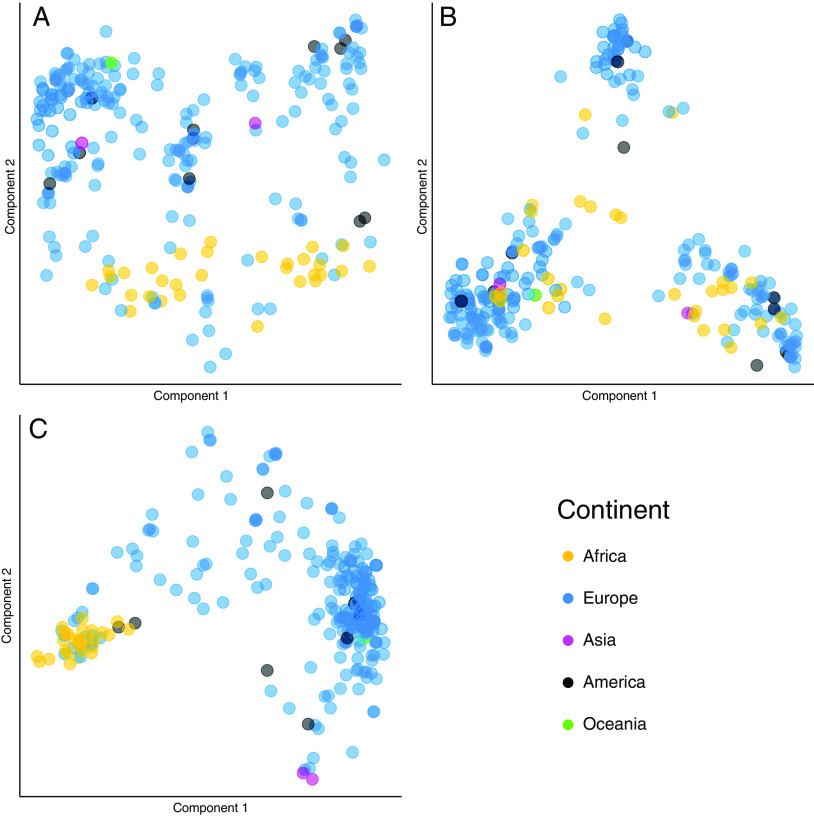
Multidimensional scaling of all CMV genomes. The figure shows multidimensional scale analysis for all CMV strains analyzed (n = 259): Each dot represents a CMV strain, and the color indicates the continent of isolation (Europe includes European and Middle Eastern genomes). The analysis was done in three scenarios: (*A*) whole genome, (*B*) conserved regions (conserved concatenome), and (*C*) multiallelic regions (multiallelic concatenome). This analysis shows an overall trend for geographical segregation for the whole genome (*A*) and the conserved regions (*C*), but not for the multiallelic regions (*B*).

To examine the relationship between CMV strains, we first analyzed whole genomes by the geographical region (continent) in which they were sampled. Geographical segregation of genomes is well described for other herpesviruses (38–41). Because CMV is known to be highly recombinant such that accurately reconstructing phylogenetic relationships and tree topology become problematic, we initially used multidimensional scaling (MDS) to analyze the genomes (Fig. 3) as dimensionality reduction techniques simply represent “closeness” of sequences and have been able to derive representations of genetic data resembling their geographic ancestry (42, 43). The resulting clustering pattern showed geographic segregation of whole genomes in the second most important dimension (component 2, *SI Appendix*, Fig. S3) with those sampled in Africa clustering away from most sequences sampled in Europe. Two sequences sampled in Asia were located among the European sequences (Fig. 3). MDS components 1, 3, and 4 which represent large variance in the data were uninformative with respect to geographical segregation of whole genomes.

This geographical split was corroborated by a phylogenetic network tree, which allows representations of shared recombination events as network splits (*SI Appendix*, Fig. S4). Sequences sampled in the Americas (all from the USA) were distributed throughout the plot. The single sequence from Oceania (Australia) appeared to resemble sequences sampled in Europe. A minority of sequences sampled in Europe clustered with sequences sampled in Africa, but not vice versa (Fig. 3). To examine this in more detail, we made use of a separate set of CMV genomes from a cohort of CMV seropositive solid organ transplant recipients (n = 11, Dataset S2) with available self-reported ethnicity data, all of whom were sampled in the United Kingdom (44). Performing MDS of these sequences along with the European, African, and Asian sampled CMV showed that CMV from transplant recipients of self-reported African and Afro-Caribbean origin but sampled in the United Kingdom clustered predominantly with strains sampled in Africa and separately from UK patients of non-African origin (*SI Appendix*, Fig. S5). This suggests that sequences cluster by host ancestry rather than simply by sampling location. The few European sampled strains that clustered with the bulk of African samples sequences are therefore likely to be from individuals of African ancestry. Our findings contrast with previously published results that failed to identify CMV population structure related to geographical origin in partial genomes (45, 46) or a subset of highly heterogenous whole genomes (11), although the two published Asian sequences have been noted as tightly clustering in an analysis of whole genomes (10).

### Conserved and Multiallelic Genomic Regions Show Distinct Patterns of Phylogeography.

Because of potential differences in evolutionary histories between conserved and multiallelic regions, we next analyzed these separately for geographic structure. MDS of the concatenated conserved genomes (concatenome) showed more distinct separation between viruses of African and European origin than the whole genome sequences, as well as clearer segregation of the Asian CMV genomes (Fig. 3 and *SI Appendix*, Figs. S4 and S5). In contrast, multiallelic regions when concatenated appeared to show minimal geographic clustering. We quantified the differences between African and European populations in conserved regions using the fixation index (Fst) which compares diversity within and between different populations. As Fst can be biased if sample sizes vary between populations (47), we randomly chose 30 African-sampled and 30 European-sampled sequences. The continent labels were then randomly scrambled to generate a null hypothesis Fst, and both steps were repeated 10,000 times to obtain true and null Fst distributions (*SI Appendix*, Fig. S6).

The results showed that the conserved regions in the CMV genome encode clear geographic (continental) differences, with a mean Fst of 0.21 (a 423% increase on the mean null Fst). Fst of the concatenated multiallelic regions was relatively weak at 0.097, which is only a 194% increase on the mean null Fst. While both Fst values were significantly different from their respective null distributions (both Mann–Whitney test *P*-values < 0.0005.), the geographic signal appears to be more enriched (423% vs. 194%) for the conserved regions of the CMV genome.

### Admixture Model-Based Estimation of Ancestry Supports Continental Population Structure.

**Fig. 4.**
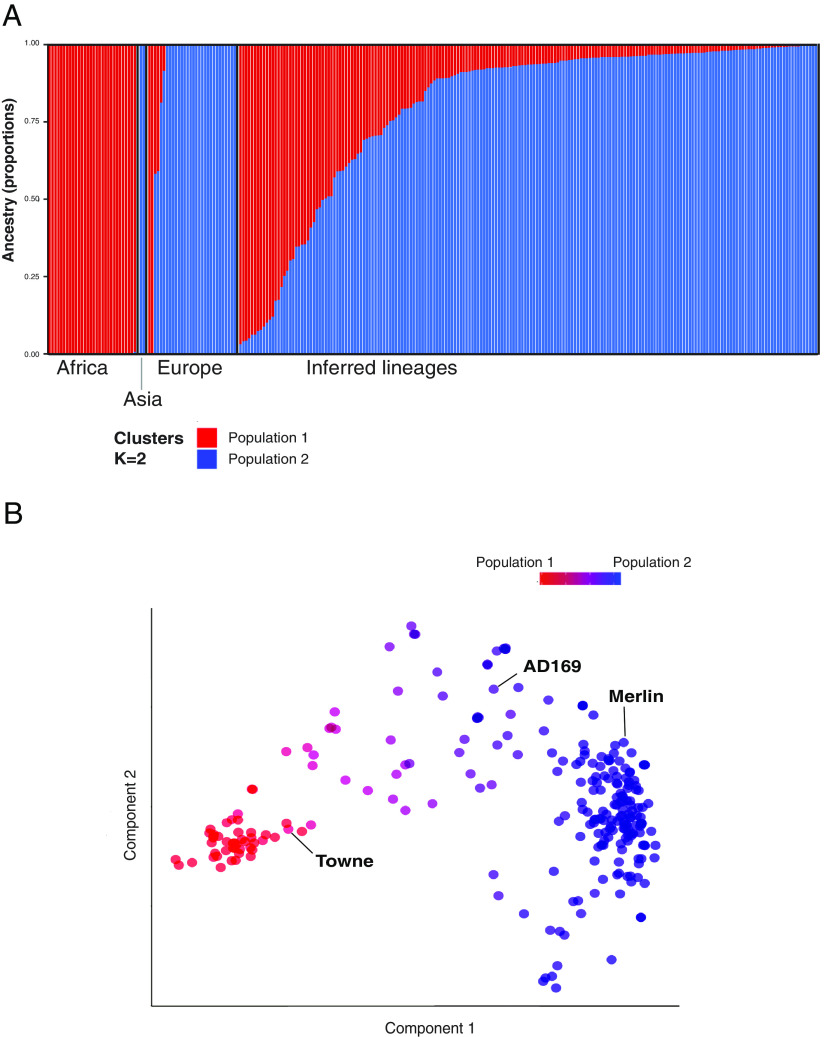
Admixture analysis in CMV’s conserved regions. Admixture inferred ancestral lineages reconstructed from CMV conserved concatenomes support evidence of geographic segregation in CMV. The admixtures derived from a representative K = 2 model of 62 sequences were projected to the remaining 197 sequences. (*A*) This plot shows admixture proportions for whole CMV dataset (n = 259 strains) grouped by continent. (*B*) The red and blue cluster components were used to color sequences in the conserved concatenome MDS. Select common reference strains have been labeled.

To further examine the segregation of conserved genomic sequences by continent and to use a model-based approach to complement the visual MDS and network phylogeny, we undertook an admixture analysis which attempts to infer the ancestral lineages and the contributions from each that gave rise to a set of modern sequences (Fig. 4) (48). Admixture analyses, like dimensionality reduction, can also be skewed by large sample size differences between groups and by heterologous sampling methodology between groups (49). To account for the former, we randomly subsampled to generate more proportionate sample sizes per continent (30 European, 30 African, and both Asian sequences), then calculated the cross-validation error (CVE) for K = 1 through 10. This was repeated for 1,000 random sample draws.

Two lineages (K = 2), one African and one Eurasian, were found to be the consensus result from the 10 lowest error models (Fig. 4*A* and *SI Appendix*, Fig. S7*A*). CMV sequences sampled in Africa from individuals of African ancestry were clearly identified by the model as being part of this African lineage, with no hint of admixing and vice versa for white Europeans. Few sequences sampled in Europe were assigned strongly to the African lineage, but in the MDS, these are likely explained as being sampled from ethnic African individuals in Europe (Figs. 3*A* and 4*B* and *SI Appendix*, Fig. S5). Many sequences also appeared as admixes and correspond well with those sequences that lie between the clusters of African and European in the MDS.

For a minority of subsampling replicates, there was a random skew toward sampling more Asian-like CMV sequences, and in three replicates, the optimal model was K = 3 clusters (*SI Appendix*, Fig. S7). Although the current data are best explained by two ancestral genomes (K = 2), the few Asian sequences (n = 2), although clustering together, will not change the admixture result for most replicates (49). Notwithstanding, there was also clustering of two Asian CMV conserved concatenomes from UK patients of self-reported Asian ethnicity (*SI Appendix*, Fig. S5 and Dataset S2) with the two Asian-origin GenBank sequences making it likely that with more genomes, three ancestral sequences (K = 3) may turn out to better represent the data.

To overcome the complexity of sample origin not necessarily reflecting virus ancestry (i.e., human migration) and to allow better delineation of population differences between African and European CMV lineages, we limited further analyses to “archetypal” sequences from each continent. Archetypal sequences were defined as those with >90% admixture proportion to a single ancestral cluster using the conserved concatenome data, in the lowest error k = 3 model. Using this definition, we identified 42 strains (12 of which were sampled in Europe) of archetypal African ancestry and 129 of archetypal European viral ancestry, which includes the reference strain Merlin. Four CMV sequences were identified as of archetypal Asian ancestry (two of which were sampled in Europe from subjects with self-reported Asian ancestry).

### Geographic Signal Is Consistent across the Conserved Regions and Sequences between Continental Clusters Have Random Patterns Ruling Out Recent Recombination.

To understand how the geographic signal is ordered across the viral genome, we identified the loci within the conserved concatenome with high (>0.5) Fst values, i.e., which showed the most distinction between African and European strains. To provide a visual representation of the continent of origin for each strain, we colored high-Fst-value African nucleotides present at consensus (>50%) in archetypal African strains red and high-Fst-value European nucleotides present at consensus (>50%) in archetypal European strains blue. Sites which were not present at consensus in ether archetypal African or European strains were colored (black) (*SI Appendix*, Fig. S8). In the archetypal African or European subset, sequences were overwhelmingly represented by the base most common to their continent. Moreover, where an African consensus base appears in an archetypal European strain, or vice versa, this appeared to be largely random. Sequences not archetypal of these three continents had complex, although apparently nonrandom, patterns of admixture.

### Multiallelic Genomic Regions Represent a Mixture of Geographic Relationships.

We next examined the apparent but weaker geographical segregation of the 74 multiallelic regions between African and European populations. As the multiallelic regions each contain variable numbers of alleles and are of different lengths, we considered each of the 74 regions separately. We performed chi-squared tests with allele distributions to evaluate whether multiallelic regions segregated with archetypal African or archetypal European strains. The results revealed strong geographic distribution of alleles for 32 of the 74 regions (*SI Appendix*, Table S1; we calculated the false discovery rate, FDR, using the Benjamini–Hochberg procedure, and we set the FDR threshold at 0.05) (50), while five showed more moderate geographic differences (0.05 < FDR < 0.3; *SI Appendix*, Table S1) with the rest showing no evidence of geographical segregation. Coloring African dominant alleles red and European-dominant alleles blue (as defined above) for the 32 multiallelic regions that segregated geographically, we observe a similar pattern to that seen for the conserved regions, with African strains largely red and European strains largely blue (*SI Appendix*, Fig. S8 *C* and *D*).

### Host Immune–Mediated Selection Is Not Obviously Responsible for Driving Geographic Segregation of African and European Strains.

To assess whether the geographic population structure observed in conserved regions of the CMV genome was due to differences in the selective pressures exerted by different host populations, we looked at the 440 most geographically informative sites (Fst > 0.5) within the archetypal subsets of European and African strains. We asked how many of these sites resulted in a different continental consensus amino acid between African and European sequences, and, if so, whether the change occurred within known B and T cell epitopes, as recorded in the Immune Epitope DataBase (IEDB) (51). Only 15% (64) of the 440 sites encoded nonsynonymous changes. Of these, 16% (10 of 64) lay within known epitopes, compared with 14% (52 of 376) of synonymous sites, providing little evidence that geographic population structure in CMV is driven by continentally unique host immune pressure.

### Alleles in Immunomodulatory Genes Tend to Maintain Similar Diversity across Continents.

We next tested whether certain gene functions were overrepresented in three classes of genomic regions: conserved, geographically segregating multiallelic, and nongeographically segregating multiallelic regions. To do this, we identified the genes lying within each region class and annotated their function, using three predefined functional groups from a published gene ontology (latency, tropism, and immunomodulation) (52). We then determined whether key functional groups were significantly overrepresented/underrepresented by region class (*SI Appendix*, Fig. S9). We found that the 74 multiallelic regions together were significantly enriched for genes encoding immunomodulatory functions with the proportion of genes with immunomodulatory function of 0.371 (37.1%), where for all genes, this proportion is 0.206 (FDR = 0.0096). This enrichment was clearer still if only the 42 multiallelic regions with no evidence of geographical segregation were considered (39.1%, FDR = 0.014). No other comparison was significant.

### Geographic and Multiallelic Genetic Differences May Impact Biological Function.

One interesting application of studying multiallelic regions is the possible association between different alleles and function. To illustrate this, we investigated glycoprotein B (gB, UL55) as an example. The reference strain Towne has been extensively used to develop vaccines and to study CMV immunogenicity and function (53), and its gB protein variant has been the basis for many vaccine candidates (54). From the admixture analysis, we identified Towne as being 79% African, while two other common reference strains, AD169 and Merlin, were 75% and 99% European, respectively. The gB (UL55) sequence is composed of three separated multiallelic regions (22, 23, and 24 in *SI Appendix*, Table S1) which are linked (Monte-Carlo chi square *P* < 0.001) in 12 possible combinations or haplotypes. In addition to showing geographically informative genetic differences from European strains in its conserved regions, Towne gB also differs in the sequence of its multiallelic regions sharing the same 22,23,24 (UL55) haplotype as only 4% of the 259 CMV genomes sequenced here (*SI Appendix*, Fig. S10). By contrast, the Merlin multiallelic region 22, 23, 24 (UL55) haplotype is shared with 32% and AD169 with 20% of the 259 genomes. Genetic differences in gB (UL55) have been mooted to underlie observed differences in cross-strain neutralization by antibodies raised against one strain (55, 56). In general, immunotherapies and drug development targeting CMV that rely on alleles that differ across geographic isolates may now require further investigation as to whether treatment effect will be advantageous to only certain human populations.

## Discussion

We have characterized the variability present in whole CMV genome sequences, including several known to be of African origin identifying both known hypervariable regions and over 40 other regions (10, 11, 13, 16, 57). Altogether, we describe 74 hypervariable regions comprising 14% of the genome, all of which are multiallelic. The remaining 86% of the genome is monoallelic and at least ten times less variable than the multiallelic regions. While previous reports have identified around 30 multiallelic hypervariable regions, they identified them by the gene in which they were located, despite in many cases, much of the gene concerned not being hypervariable and thus subject to different constraints on recombination and diversity. In contrast, our data precisely delineate the nucleotide coordinates of variable and conserved regions, using an unbiased and consistent assignment model. This has allowed the identification of 17 multiallelic regions that cross gene boundaries, and five are entirely in noncoding or in otherwise unassigned. For each of the 74 regions, our statistical approach also returns the number of alleles that most parsimoniously fit the data, leading to between two and eight alleles per region (*SI Appendix*, Table S1). In some cases, for example, UL146 and UL144, the number of alleles we identify differs from previous numbers reported (58, 59). However, previous estimates were determined largely by visual inspection of whole-gene phylogenetic trees, a process which resulted in differences in reported allele numbers not only from us but between other authors (for example, for UL55), with, on occasion, some alleles not being reliably distinguishable (16, 60–62). The objective mathematical approach we have adopted provides clear and reproducible multiallelic region boundaries and allele numbers, properties which will have advantages for standardizing genotyping nomenclature.

In contrast to previous reports (10, 11), we observe clear geographical segregation of CMV genomes with evidence for African, European, mixed, and possibly Asian genotypes. From an evolutionary viewpoint, geographical segregation of genomes is well described for other human herpesviruses, including the herpes simplex virus (HSV-1), varicella zoster virus (VZV), and Epstein–Barr virus (EBV) (38–41, 63) and is therefore not surprising for CMV. Our data suggest that most geographically informative SNPs in CMV are in the monoallelic genomic portions and like the rest of the genome are under purifying selection (10). Unlike EBV, in which host immune selection drives local adaptation of the virus to different human host populations, shaping the pattern of genetic diversity (64), we find no evidence that selection within immunogenic regions of the genome is a dominant driver of the observed genetic geographical differences. Instead, we postulate that genetic drift and bottleneck events such as founder viruses are plausible explanations for the population structure observed in CMV. If this is the case, there remains some difficulty in establishing the direction and date of split for European and African CMV populations due to the low association between sampling date and distance from phylogenetic tree root and a mutation rate that has proven difficult to determine for double-stranded DNA viruses (65). The difficulty in determining mutation rates is likely to be, in part, a result of CMV’s longstanding free recombination within these geographically isolated pockets. As 13 multiallelic regions were found to contain alleles unique to Europe while the opposite was not seen for African viruses, this could be taken as evidence supporting a European origin of CMV, where Africa has restricted diversity. However, this is likely more simply explained as an artifact of the differences in size and sampling heterogeneity of our available genomes.

The finding of African-clustering CMV strains in patients self-reporting as being of Afro-Caribbean ethnicity, many of whom presumably have not lived in Africa, can potentially be explained by early acquisition of CMV from family members and by assortative mating of racial groups. A similar effect has been observed for VZV in subjects of Afro-Caribbean origin growing up in the United Kingdom (41). In African countries, most children are CMV positive by their first birthday (33, 66, 67). Early Infection, most likely acquired from maternal or sibling transmission (68), may explain why subjects of African origin living in Europe test positive for strains that cluster with known African strains. This would date the split of African and European CMV strains to at least 500 y ago, the time at which the first African slaves were transported to Europe (69). Although the actual separation is likely to be much older given even the highest estimates of CMV’s mutation rate (70), or borrowing from rates presumably more accurately estimated for a similar virus, HSV1, for which there are ancient genomes available (71). With greater mixing of populations and the pervasive genome-wide recombination that occurs in CMV, we see evidence for increasing numbers of hybrid strains including some of the reference strains, for example, Towne and AD159 (Fig. 4). This serves to further muddy insights into the phylogeographic origins of currently circulating strains of CMV, and elucidating these undoubtedly requires additional and more granular worldwide sampling, as well as the inclusion of ancient CMV genetic material if these can be found.

While in 32 of the multiallelic regions, the alleles, like the conserved regions, are different or differently distributed between geographical regions, most notably countries with predominantly European or African populations, from which most strains originate, the majority (42) show no evidence of geographical distribution, but instead appear to maintain the full allele palette in these genome portions across both continents and at similar frequencies. This effect has previously been observed in RNA viruses (72, 73). Initial studies into CMV virion envelope complexes, linking variants to function, have reported that allele differences can modulate virus cell tropism (74, 75). Similar examples from the hepatitis C virus and HIV have shown genotypic differences to affect viral compartmentalization (76), while certain hepatitis B virus genotypes appear to be associated with chronic infection (77). From our analyses, the nongeographically segregated multiallelic regions were significantly enriched for genes encoding immunomodulatory functions. For example, region 6 which shows no geographical segregation encodes a portion of the nonrecombinant haplotype RL11D block, RL11, RL12, RL13, UL1, UL2, and UL4, all of which are proven or predicted to be virion membrane glycoproteins (52). In addition, RL11D (region 6) variability has been suggested to be critical for the adaptation of CMV to different primate species (11). Of interest, many of the variable multiallelic regions correspond to regions previously identified as being in local linkage disequilibrium and thus not affected by the pervasive recombination occurring throughout the more conserved regions of the genome (11). Taken together, our data strongly support the likelihood that CMV genome is the result of two distinct evolutionary forces, genetic drift occurring in segregated viral populations and so-called frequency-dependent balancing selection (78), a form of adaptation that maintains preexisting diversity in the face of genetic drift.

This granularity of CMV genome analysis allows deeper insights into how genome might be related to function. Following Towne gB + MF59 adjuvant vaccination, antibody titers to the antigenic AD2 region have been shown to correlate with better protection against postrenal transplant CMV viremia (54–56). Baraniak and colleagues also showed that only ~50% of individuals vaccinated with Towne gB had detectable AD2 antibody response against gB peptides in an assay derived from the AD169 laboratory strain. The AD169 gB AD2 allele (region 24, UL55) differs from that found in Towne gB (*SI Appendix*, Fig. S8). Since AD2 antibodies are not broadly reactive (54), there is some question about what the Towne gB vaccine antibodies are recognizing within the AD169 gB AD2 peptides. Multiallelic region 24 (gB/UL55 AD2) also segregates differently between African (Towne-like) and European-like (AD169 and Merlin) viral populations. This together with the finding that the Towne gB conserved region carries predominantly African-segregating SNPS raises the possibility that a vaccine based on the Towne gB sequence might not confer cross-protective immunity against European strains.

Notably, the CMV pentamer complex, which is being developed as part of an alternative multiantigenic vaccine using the Merlin strain, contains no multiallelic regions and may therefore be more tractable than gB (79). However, geographically related differences are still present and potentially need evaluation. For example, Q35K which segregates with African strains and L40P which segregates with European strains are both present within a known B cell–neutralizing epitope in the pentameric complex UL130 protein (IEDB ID: 142031).

These analyses are subject to limitations, the clearest of which is the potential biases related to the available sequences and the samples from which they were derived. First, the representation of genomes was heavily skewed toward Europe. Second, the European samples were typically collected from unrelated patients for clinical purposes, whereas African samples were obtained from study participants in southern and eastern Africa (Zambia, Uganda, and Kenya), many of whom were HIV coinfected. African-clustering sequences that were sampled in Europe likely reflect divergent sampling of unrelated individuals and may to some extent mitigate the bias of African strains. However, we had only two Asian strains collected in the early 2000s and no strains from South America or many other parts of the world. Until these gaps are closed, our conclusions must remain incomplete. Seventeen African genomes were reconstructed from samples containing mixed CMV infections, where generating consensus genomes has typically been challenging. However, HaRold, the program we used to reconstruct haplotypes (31), performs with high accuracy in validation exercises using simulated and real mixtures of CMV genomes containing known sequences. The lack of homology between some alleles within a multiallelic region could be a limitation in constructing alignments for HMM. However, even for the most divergent alleles, the bordering 5′ and 3′ sequences are identical, a factor that mitigates this potential constraint. Finally, when considering B and T cell CMV epitopes, we are limited by epitopes included in the IEDB database which are largely generated for European strains. However, since most of the viruses analyzed here were European, the conclusion that most nonsynonymous differences from African strains do not lie within epitopes is likely to be true.

## Conclusions

Our findings provide several insights into the genomic landscape of CMV. First, we identify and precisely delineate 74 discrete variable regions which consist of multiple alleles, showing that the rest of the genome (86%) is monoallelic. We identify that CMV genomic evolution is shaped by two distinct processes: likely genetic drift occurring within geographically distinct populations and balancing selection which counteracts genetic drift to maintain similar diversity in variable multiallelic regions irrespective of geographical location. We identify that variable regions under balancing selection are enriched for key CMV properties, highlighting that better characterization of diversity in these regions is likely to be important for understanding CMV biology and control. Our results provide a genomic roadmap to enable studies of how variation across the CMV genome interacts to cause clinical disease. At the same time, the data raise questions about how geographical differences arose and the direction of spread from one region to another. The answers to these questions will require further sampling of geographically diverse whole CMV genomes, CMV sequence data from ancient samples, or both. Finally, the data highlight that the geographical and allelic differences between proteins being trialed as potential vaccines need to be considered when designing vaccines. Our findings raise the possibility that vaccines based on strain-specific gB or other viral antigens may fail to induce sufficient cross-protection globally against circulating variants.

## Materials and Methods

### Data Retrieval.

A Python script using Biopython (80), specifically the Entrez module, was used to access the SRA and NCBI nucleotide databases for sequence information and extract country and continent assignment for sequences.

### Sequence Assembly.

SRA sequences for Zambian CMV genomes were downloaded using the SRA toolkit and assembled using an in-house de novo assembly pipeline, which involves contig generation, optimal reference identification, scaffolding onto the reference sequence, and subsequent iterative mapping of NGS reads on the genome scaffold. These were then subject to haplotype reconstruction and relevant consensus sequences determined.

Ugandan sequences and historical clinical sequences of known ethnicity were also de novo assembled. Kenyan sequence data were assembled to a reference sequence using an in-house pipeline using the strain. Sequence positions with less than 10-read depth were labeled as n.

### Haplotype Reconstruction.

Possible mixed infections were investigated with HaROLD, a tool for reconstructing haplotypes using co-varying variant frequencies in a probabilistic framework (31). HaROLD takes the bam files obtained from the assembly step (as explained above) and then reconstructs the optimal number of haplotypes for each sample. Haplotypes’ sequences are then checked by reconstructing phylogenetic trees and are considered distinct if they have >2,000 bp differences. We reconstructed a total of 17 haplotypes from eight samples (1 to 3 haplotypes per sample). In line with the approach taken in de novo assembly, we ignored haplotypes with an average read depth of less than 10 bases (haplotype frequency * mean read depth).

### Multiple Sequence Alignment.

Multiple sequence alignments were obtained using MAFFT v7 (81), particularly variable sections were realigned using MUSCLE (82) and checked manually. Sequence alignments were viewed in the lightweight alignment viewer AliView (83). Alignments relative to a reference strain were only used to generate the heterozygosity per reference position calculation; these were generated using MAFFT with the “—add – keeplength” options, which allowed SNPs to be called based on differences to the reference Merlin (Refseq accession: NC_006273.2).

### Measures of Sequence Diversity.

Heterozygosity was generated using an in-house R function, using the following calculation. h is heterozygosity for a given polymorphic site with I alleles, such that the sum of all allele frequencies *P* equals 1. N is the number of sequences in the sample. Summing over all segregating sites S in an alignment, we get the sum of site heterozygosity π.

$$h=\frac{n}{n-1}\left(1-\sum_{j=1}^{S}p_{i}^{2}\right)\pi =\sum_{j=1}^{S}h_{j},$$

### Multidimensional Scaling.

Pairwise distances were calculated using the dist.dna() with the nucleotide–nucleotide substitution matrix “TN93” (84) and with pairwise deletion by way of the R package Ape v.5.4 (85). Multidimensional scaling much like principal component analysis is a method to attempt to simplify complex data into a more interpretable format, by reducing dimensionality of data while retaining most of the variation. In a genomics context, we can use this on pairwise distance matrices, where each dimension is a sequence with data points of n-1 sequences pairwise distance. This allows us to observe patterns of population structure as “clusters”. MDS was implemented using the cmdscale() function with pairwise deletion in R (86).

### Phylogenetic Reconstruction.

Phylogenetic relationships of multiallelic and example conserved regions in Fig. 2 were constructed from nucleic acid sequences in IQ-TREE (87). Using a maximum likelihood GTR substitution model with a discrete gamma heterogeneity model (88) and 1,000 rounds of bootstrapping, we attempted to root the CMV homologous genome to the most suitable ex-CMV taxa—chimpanzee CMV (accession AF480884); however, this outgroup was too far removed with distance >5, so unrooted trees were preferred. Trees were visualized using Figtree (89).

For the whole genome and concatenome phylogenetic analysis where CMV is known to recombine freely, a Neighbor-net split phylogenetic network analysis was undertaken using Splitstree version 4.1.5 (90). Nondefault options chosen were HKY85 distance matrix, with equal site rate variation. Both terminal repeat regions were trimmed from alignments (although they had negligible impact) before analysis.

### “F_st”_ F Statistics.

For calculating an F_st_-like statistic from sequence data, we can use the sum of site heterozygosity across a locus to produce γ_st_. Here, π_T_ is calculated as above using all samples in an alignment, and π_S_ is an average of the same calculation for each subpopulation separately (91).

$$\gamma_{\mathrm{ST}}=\frac{\pi_{T}-\pi_{S}}{\pi_{T}},$$

Only sites with greater than 5% minor allele frequency were considered. To account for uneven African and European populations, either when defined by sampling location or when considering those sequences that are archetypally (90%> in admixture analyses) African or European, we repeatedly subsampled the European population to be equivalent to the number of African sequences (1,000*) and took the mean of the site Fst’s.

When we multiply bootstrap sampled the 30 African and 30 European sequences, the mean number of pairwise differences for sequences within each population were determined, as well as the mean number of pairwise differences across all sequences. This can be used to estimate Fst in an efficient manner for multiple bootstraps (92).

### Chi-Squared Analysis of Allele Proportions.

For each region, the allele assignments from HMMcluster were grouped by origin into African and European allele frequencies as observed in the admixture archetypal strains, which we tested for significant differences using a chi-squared test of independence with the base R function chisq.test over allele frequencies. From the European data, we generated expected allele frequencies which were compared against observed allele frequencies from Africa. Using Benjamin–Hochberg adjusted false discovery rate (FDR) < 0.05 as cut-off, we determined whether a multiallelic region was “geographic” or “pervasive”.

### HMMcluster—Sequence Clustering by Optimal Hidden Markov Models.

We implemented a maximum likelihood allele assignment model in java on a hidden Markov model statistical framework. Briefly, this approach considers the genomic alignment as a set of contiguous blocks; within each block, the model instantiates by perfectly representing each sequence as its own HMM, and this results in the highest likelihood but with an excessive number of parameters. Then, the model considers the optimal way to combine HMMs to keep the highest likelihood, i.e., 259 models to 258, and continues to iterate with a greedy stepwise algorithm until only a single HMM is reached. A single HMM most poorly represents each sequence, such that the likelihood is lowest, but it uses the minimal number of parameters. To balance this likelihood and parameter problem, a typical approach is to appeal to the Akaike information criterion, and we use this here to identify the most parsimonious representation of a given genomic segment.

Consider a set of sequences $\left\{x_{1j},x_{2j}\cdots x_{\mathrm{nj}}\right\}$ where $x_{\mathrm{ij}}$ is the base found at position i in sequence j , where there are N positions in sequence ( 1≤i≤N ) and M sequences ( 1≤j≤M ). Each hidden Markov model, not considering insertion and deletion states (of which we ignore insertion states), is defined as a series of match states which are represented by the probability of the emissions from that state. That is, the hidden Markov model is defined by where $p_{i}\left(x\right)$ is the probability that match state i emits base x , and $\sum_{k}p_{j}\left(x_{k}\right)=1$.

$$\left\{\left\{\begin{array}{c} p_{1}\left(x_{1}\right)\\ p_{1}\left(x_{2}\right)\\ \vdots \end{array}\right\},\left\{\begin{array}{c} p_{2}\left(x_{1}\right)\\ p_{2}\left(x_{2}\right)\\ \vdots \end{array}\right\},\left\{\begin{array}{c} p_{3}\left(x_{1}\right)\\ p_{3}\left(x_{2}\right)\\ \vdots \end{array}\right\}\cdots \right\}.$$

In this case, the probability that sequence j would arise from this hidden Markov model is equal to $\prod_{i}p_{i}\left(x_{\mathrm{ij}}\right)$ , or the log likelihood is given by $\sum_{i}\log p_{i}\left(x_{\mathrm{ij}}\right)$ . The total log likelihood for the set of M sequences is then equal to $\sum_{j}\sum_{i}\log p_{i}\left(x_{\mathrm{ij}}\right)=\sum_{i}\sum_{j}\log p_{i}\left(x_{\mathrm{ij}}\right)$.

If we consider a given location i and imagine that at this site, $m_{i1}$ of the sequences have base $x_{1}$ , $m_{i2}$ of the sequences have base $x_{2}$ , etc, with $\sum_{k}m_{\mathrm{ik}}=M$ , then we can sum over identities of bases rather than sum over sequences, and the log likelihood becomes $\sum_{i}\sum_{k}m_{\mathrm{ik}}\log p_{i}\left(x_{k}\right)$ . Therefore, the best (i.e., maximum likelihood) values for $p_{i}\left(x_{k}\right)$ is equal to the fraction of the sequences that have base $x_{k}$ at that position, that is, ${\hat{p}}_{i}\left(x_{k}\right)=\frac{m_{\mathrm{ik}}}{M}$ , such that the highest likelihood of the set of sequences is given by $\sum_{i}\sum_{k}m_{\mathrm{ik}}\log\frac{m_{\mathrm{ik}}}{M}$.

We implemented the model to look for evidence of population structure initially within 200-bp genome slices. Identified loci were refined by maximum likelihood, and any overlapping regions concatenated, and again start/stop positions refined by maximum likelihood.

### Recombination Analysis.

Genome sequences were examined for evidence of systematic recombination events using the Recombination Detection Program (RDP) version RDP5.5 with the maximum likelihood tree option (93). The RDP software includes a suite of recombination-detecting algorithms where we used seven, namely phylogenetic (RDP, BOOTSCAN, and SISCAN) and substitution (GENECONV, MAXCHI, CHIMAERA, and 3-SEQ) methods to generate evidence of recombination. A region was considered to have evidence of recombination if at least 5 methods in RDP5 were significant (*p*-value ≤ 0.05 after Bonferroni correction).

### Population Structure.

Population structure was analyzed in an unsupervised fashion with Admixture 1.3.0 (48). Alignments were converted to VCF format using snp-sites (94), and sites with minimum allele frequency < 5% were trimmed. Sequences were randomly subsampled to generate more proportionate sample sizes per continent (30 European, 30 African, and both (2) Asian) 1,000 times. For each of the 1,000 sample draws, admixture was run for a k ranging from 1 to 10 with 20-fold cross-validation. As recommended in the admixture manual, we thinned the markers according to the observed sample correlation coefficients using the plink argument “—indep-pairwise 50 10 0.1”. Analyses were visualized in R.

### Identifying Epitopes.

Known CMV B and T cell epitopes were downloaded from IEDB (51) and then mapped to Merlin reference strain genomic coordinates by tblastn (95). Predicted epitopes were ignored. cmvdrg (96) was used to identify which variants are synonymous or nonsynonymous when translated. Sites with less than 10% variants in either African or European populations were ignored. As sites with low variability can still exhibit high Fst values, we limited the analysis to sites where the consensus base was different between the archetypal African and European sequences; this removed 11% of sites. This allowed variant sites to be analyzed together from geographical, immune, and protein effect frames of reference.

## Supplementary Material

Appendix 01 (PDF)

Dataset S01 (XLSX)

Dataset S02 (XLSX)

## Data Availability

The HMMcluster program is freely available under the MIT license, at https://github.com/ucl-pathgenomics/hmmcluster (97). The accession identifiers (GenBank or SRA accessions) for the 259 genomes included in the main analysis and the genomes of those patients with self-reported ancestry are available in Datasets S1 and S2. The full alignment and full-resolution phylogenetic trees are also available at https://github.com/ucl-pathgenomics/HCMV_resources_public (98). Previously published data were used for this work [Accession numbers (GenBank or SRA) for each genome are available in *SI Appendix*, Tables S1 and S3]. All study data are included in the article and/or supporting information.
