# Supplementary material for: Genomic and geographical structure of human cytomegalovirus
Source: Proc Natl Acad Sci U S A. 2023 Jul 17;120(30):e2221797120. doi: 10.1073/pnas.2221797120 (PMC10372631; doi:10.1073/pnas.2221797120)
Supplement: Supplementary file 1 — Appendix 01 (PDF) [file pnas.2221797120.sapp.pdf]

Supplementary figures

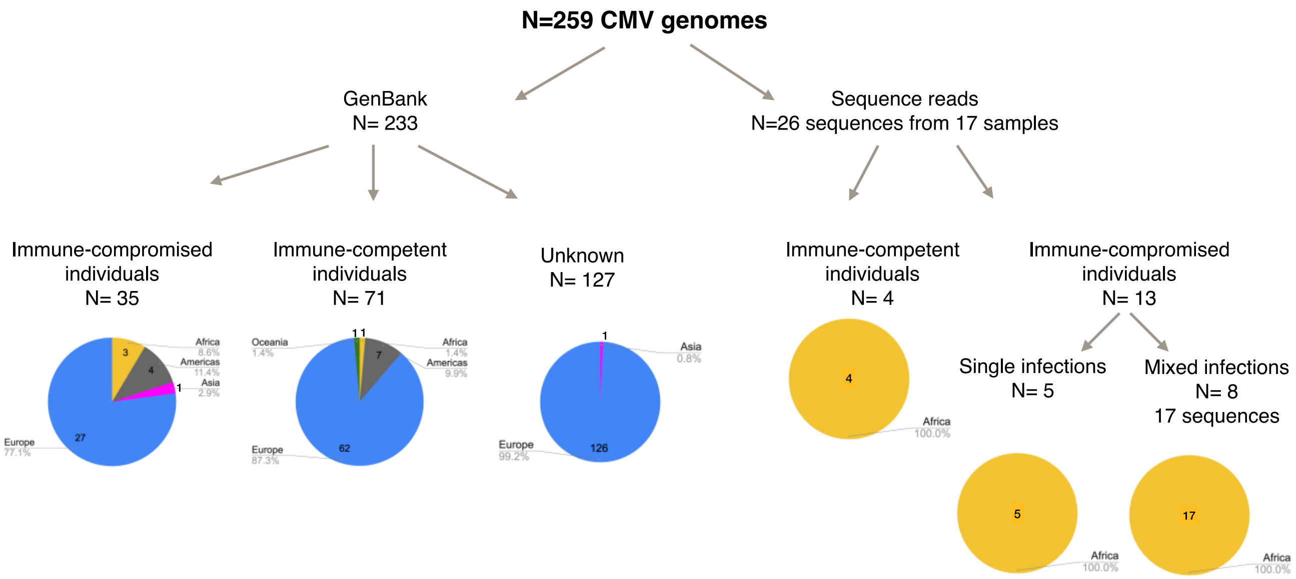

Supplementary Figure 1. Summary of the CMV genomes dataset

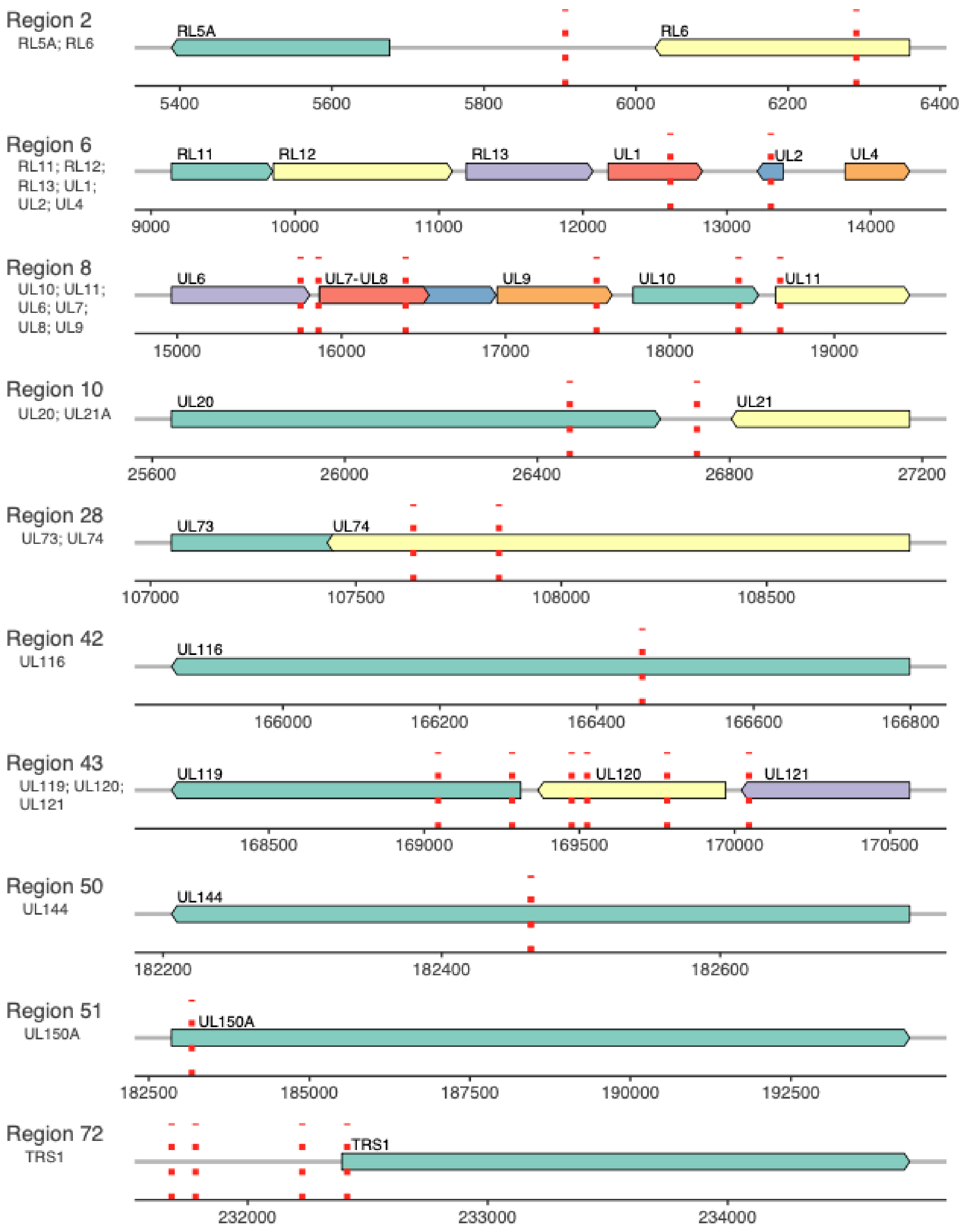

Supplementary figure 2. Multi-allelic regions with recombination events. The figure shows the 10 multi-allelic regions with evidence of recombination. For each region we show the genomic map with NC_006273.2 coordinates, the open reading frames, and the estimated recombination breakpoints (dashed red lines).

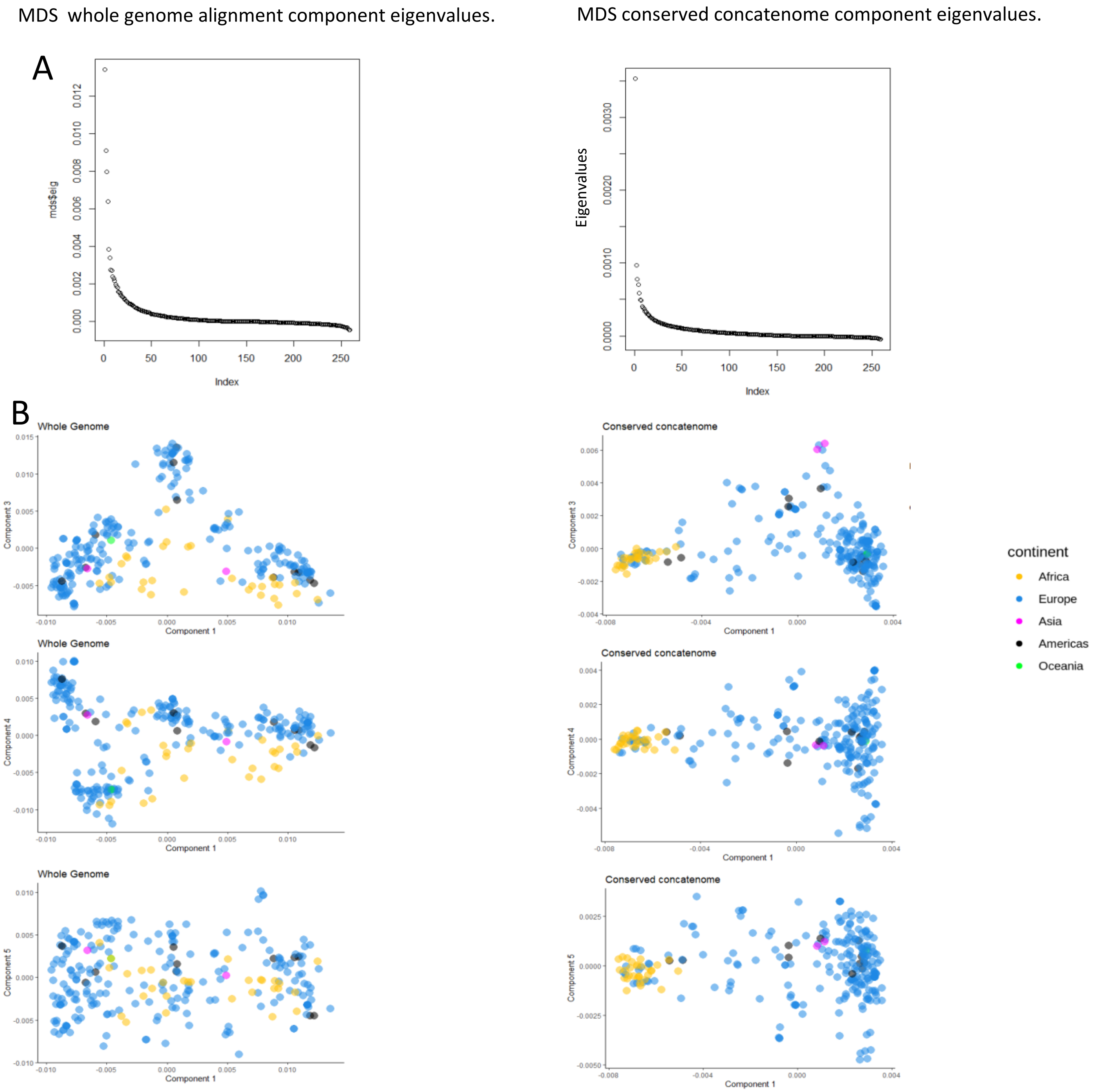

Supplementary Figure 3. Multi-Dimensional Scaling at more than two components. We set out to determine if other top components contributed towards geographical population structure. A) MDS eigenvalues representing the contained amount of variance in each component. Left – Whole Genomes: components 2, 3 and 4 encode circa 50% the variability of component 1. Right - conserved concatenomes: component 1 accounts for most of the variability which describes African-European differences. B) For whole genomes (Left) and conserved concatenomes (right), component 1 is plotted

against components 3(top), 4 (middle) and 5 (bottom). Components 3 to 5 in the whole genome appear ambivalent to geography. Components 3 in the conserved concatenome may cluster Asian sequences, components 4 and 5 appear ambivalent to geography.

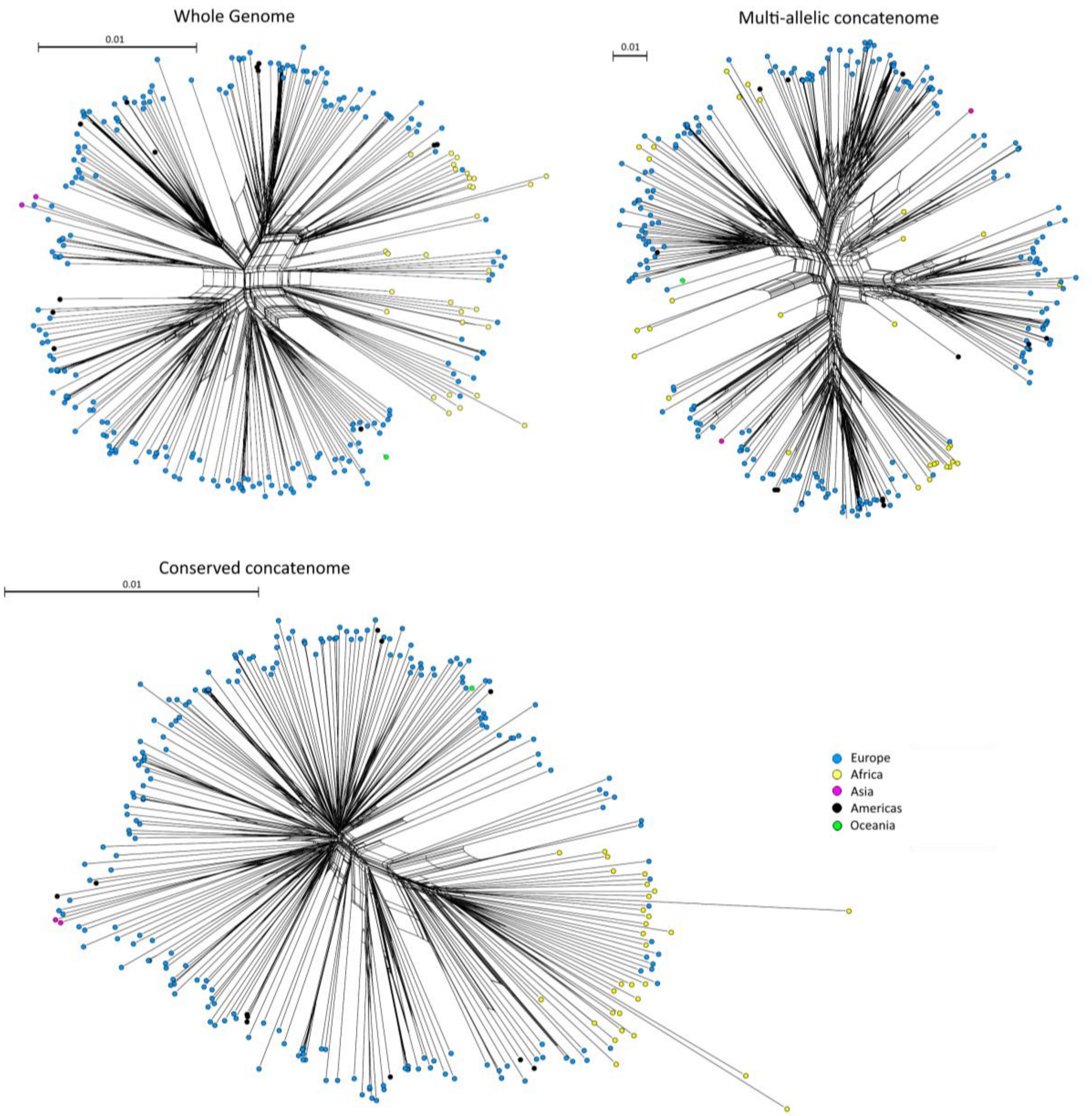

Supplementary Figure 4. Split network phylogeny of CMV. A) Whole genome sequences B) multi-allelic concatenomes. C) Conserved concatenomes. Whole genomes and conserved concatenomes cluster by continent (Africa, Europe and Asia). Multi-allelic regions as a whole show no geographical segregation. Geographic origin is indicated by node colour (see legend).

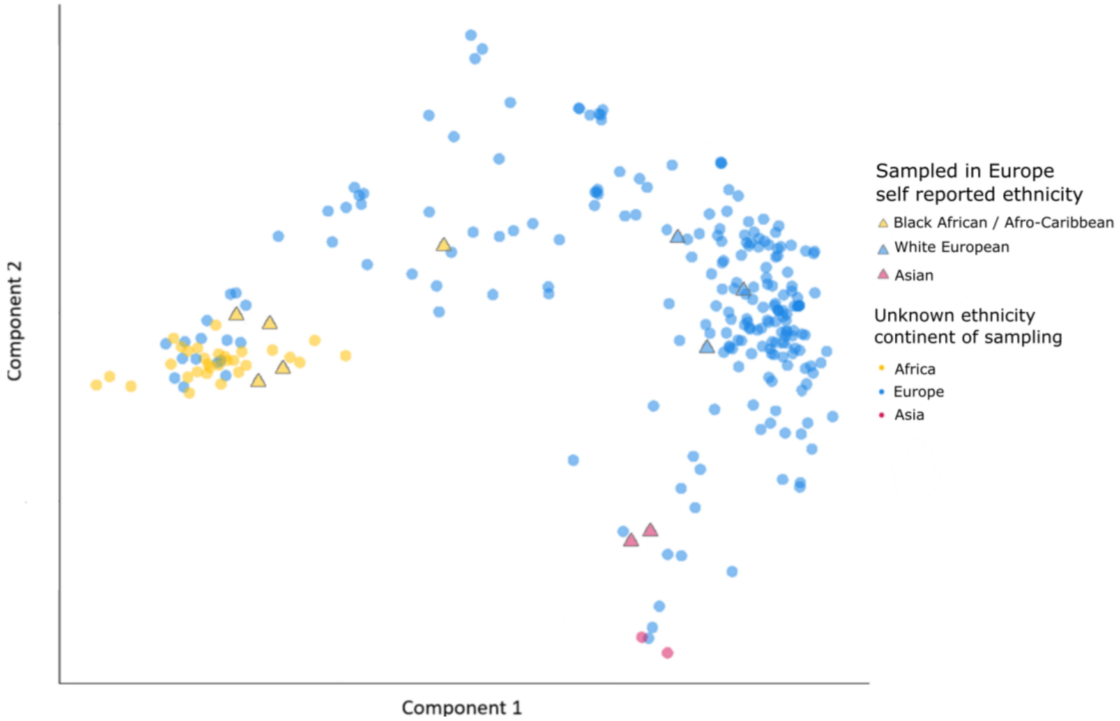

Supplementary Figure 5. Conserved concatenome MDS of Africa, Asia, and Europe-sampled sequences, including concatenomes from seropositive patients of known self-reported ethnicity. Patients with ethnicity data were all sampled and sequenced in Europe, their concatenomes are denoted by triangles. This shows continental clustering of African, European and Asian strains.

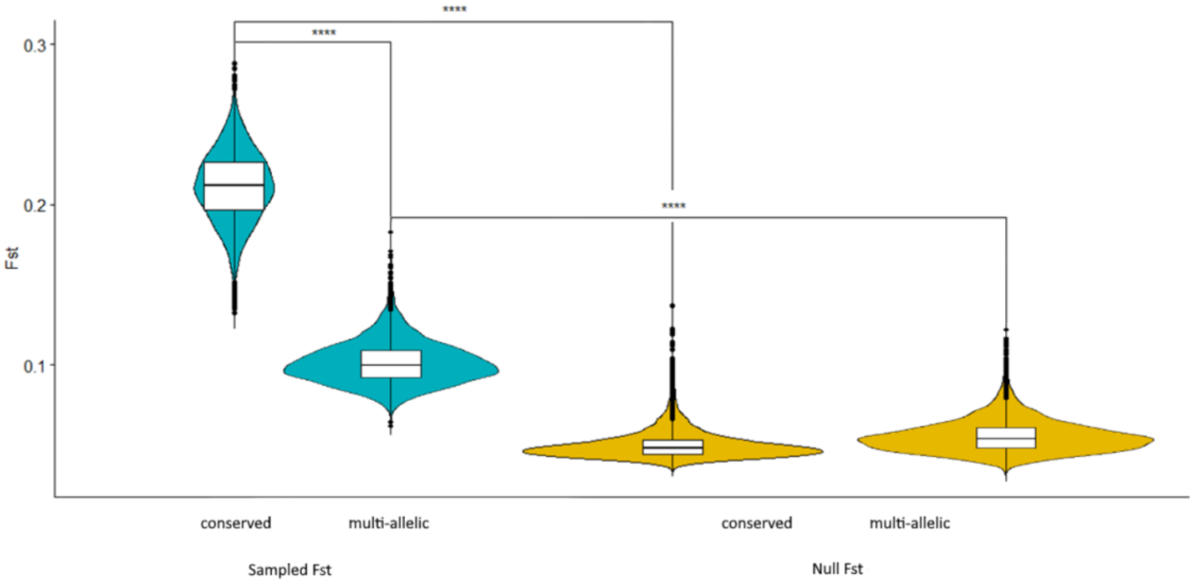

Supplementary Figure 6. Geographic signal is significantly enriched in the conserved concatenatome. Fst distributions of 30 randomly sampled African and 30 European sequences repeated 10,000 times to obtain Fst distribution for a geographically segregation ("Sampled Fst"). These were compared against randomly selected sequences for which the continent labels had been randomly scrambled 10,000 times ("Null Fst"). For conserved regions the mean Fst is 0.2115 (423% the null Fst mean). For multi-allelic regions the mean Fst is 0.0972 (194% the null Fst mean). Conserved regions therefore are relatively more enriched for geographic SNP's. Statistical analysis (independent 2-group Mann-Whitney U Test) **** indicates $p < 0.00005$.

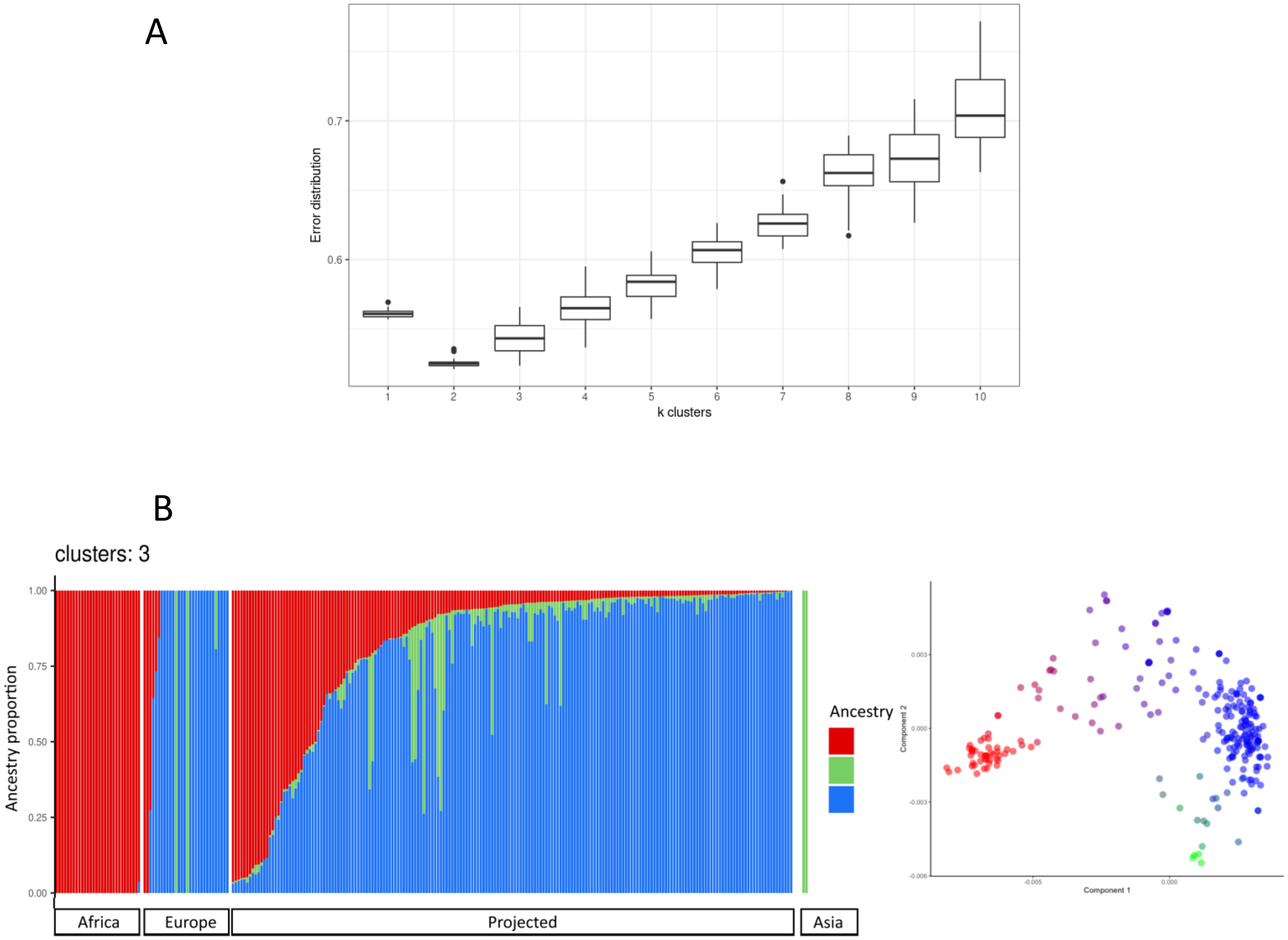

Supplementary Figure 7. Additional Admixture results. Repeat samples of the full alignment to 30 African, 30 European and 2 Asian CMV conserved concatenomes, for which Admixture analysis was run from K=1 to K=10 with 20 rounds of cross validation for determining model error. **A)** for 1000 random samples, the boxplot distribution of error values per K shows K=2 i.e. two ancestral genomes, to be the consensus optimal model. **B)** For a minority of samples the optimal description is K = 3 for which a representative admixture and MDS projection is shown. It appears likely that with further sampling of global CMV genomes the number of ancestral genomes contributing to population structure will increase to at least three.

A - Conserved Concatenome high Fst Sites

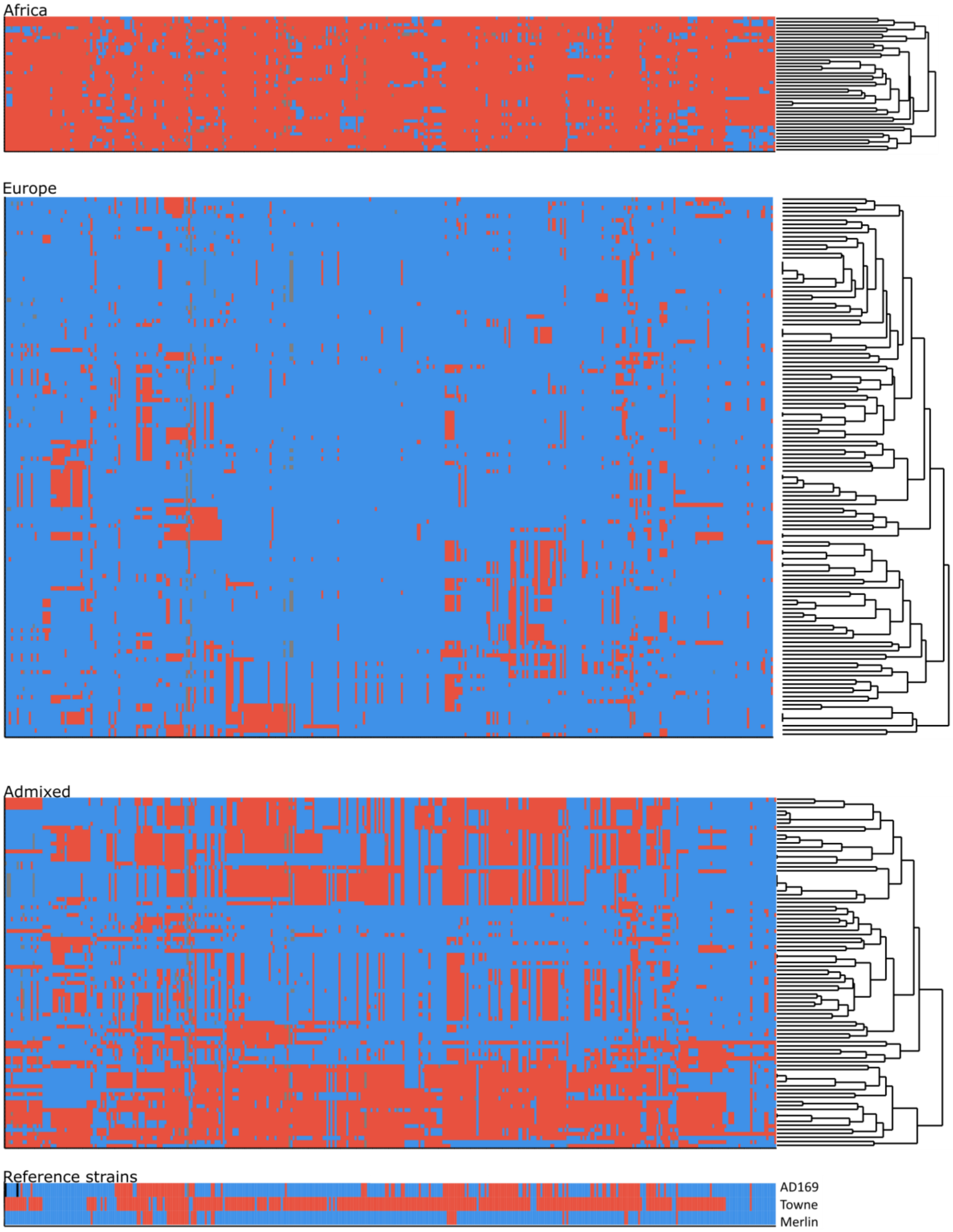

B - Geographically segregating multi-allelic regions

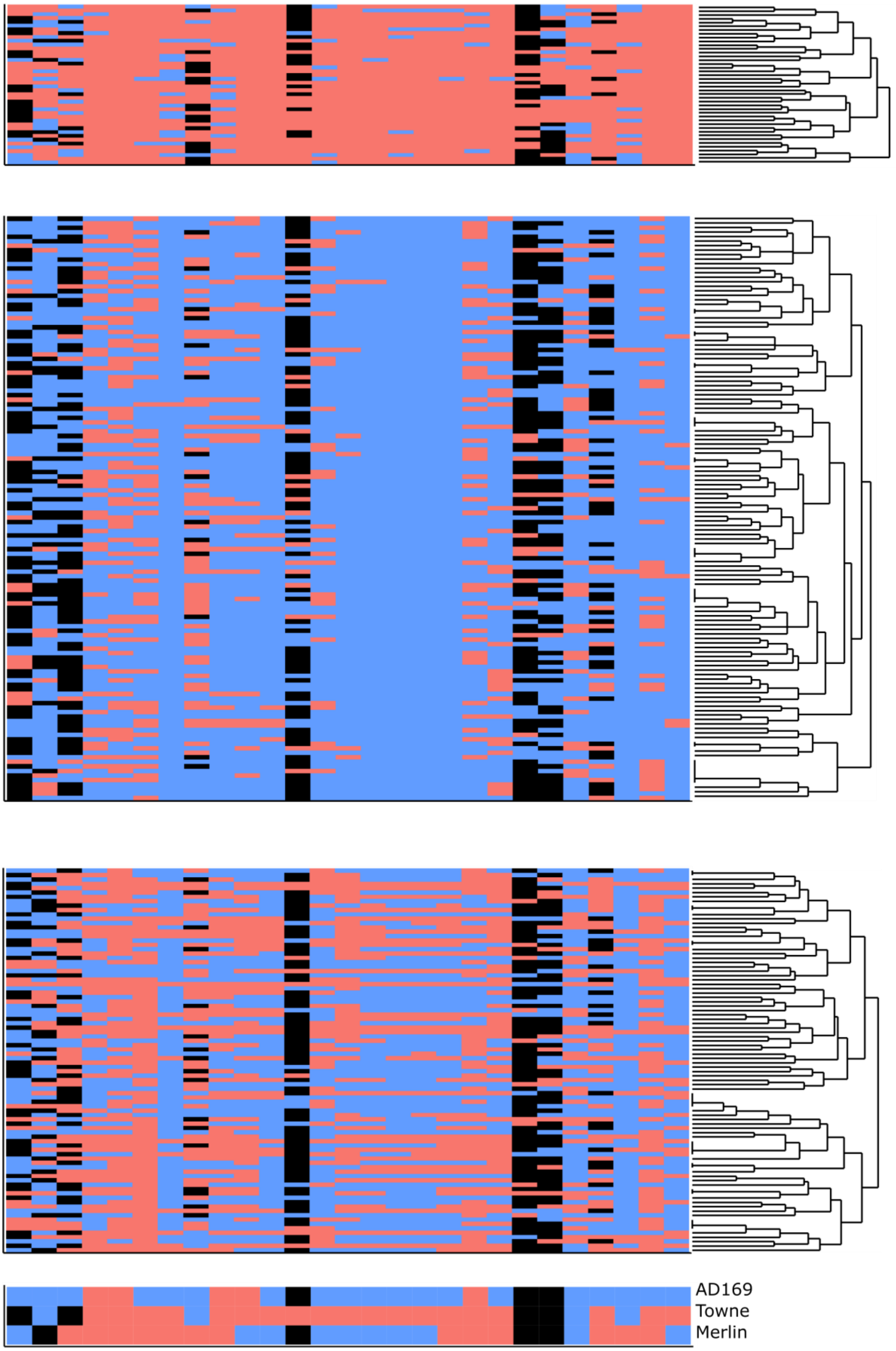

Supplementary Figure 8. Sequences are coloured by whether they contain the consensus European (blue) or consensus African (red) base, or neither (black) per site or allele. **A)** In high (>0.5) Fst conserved concatenome sites, African archetypal sequences appear dominated by red, vice versa for archetypally European CMV. Those lying between the continental cluster are mixed. In all three cases the mixtures or red with blue are not randomly distributed and may reflect unidentified sub populations. **B)** as in A but with multi-allelic geographic regions and colouring by consensus allele.

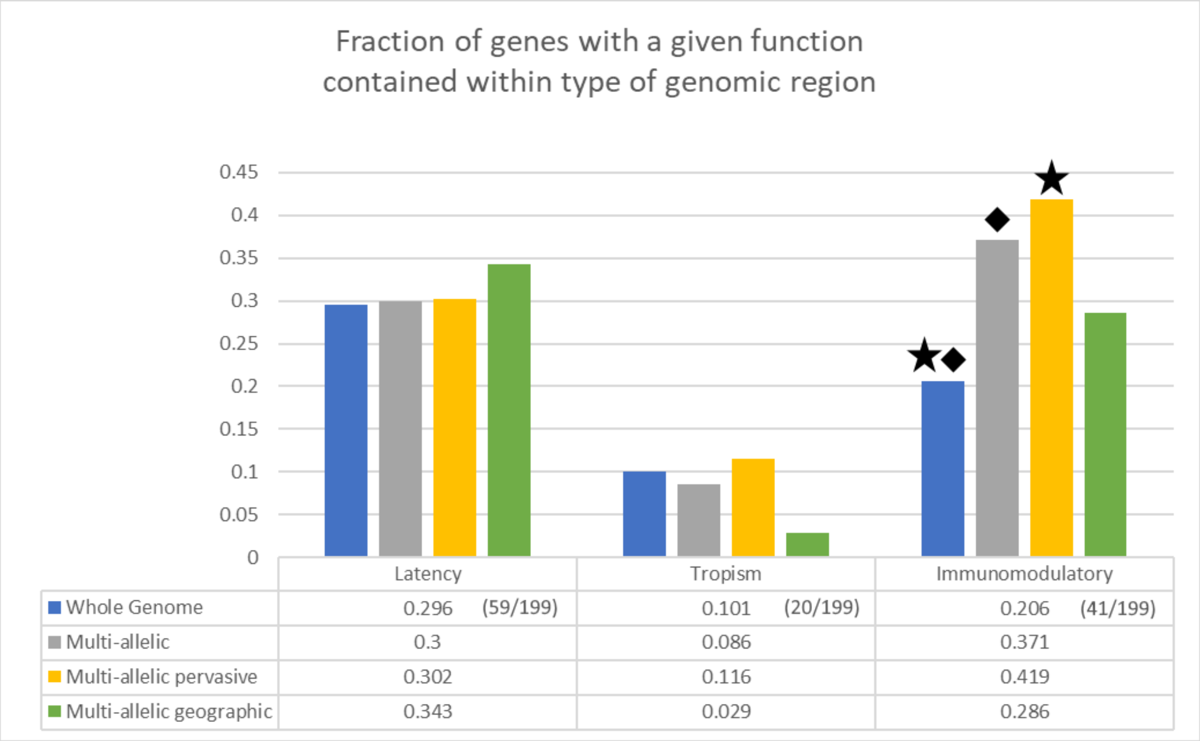

Supplementary Figure 9. Multi-allelic regions in general, and in particular those similar across Europe and Africa are enriched for Immunomodulatory products. For each genomic region, the proportion of genes or noncoding RNA's with a defined biological function as defined in (Van Damme and Van Lock, 2014). Star and diamonds indicate significantly different distributions as determined by chi squared test of independence (False discovery rate (FDR) < 0.05 (Benjamini and Hochberg, 1995)). Multi-allelic regions in general, and particularly those pervasive across continents are significantly enriched for Immunomodulatory, compared to their abundance in conserved regions or the global proportion. Functional keywords were assigned as in the reference by experimentation or proposed function.

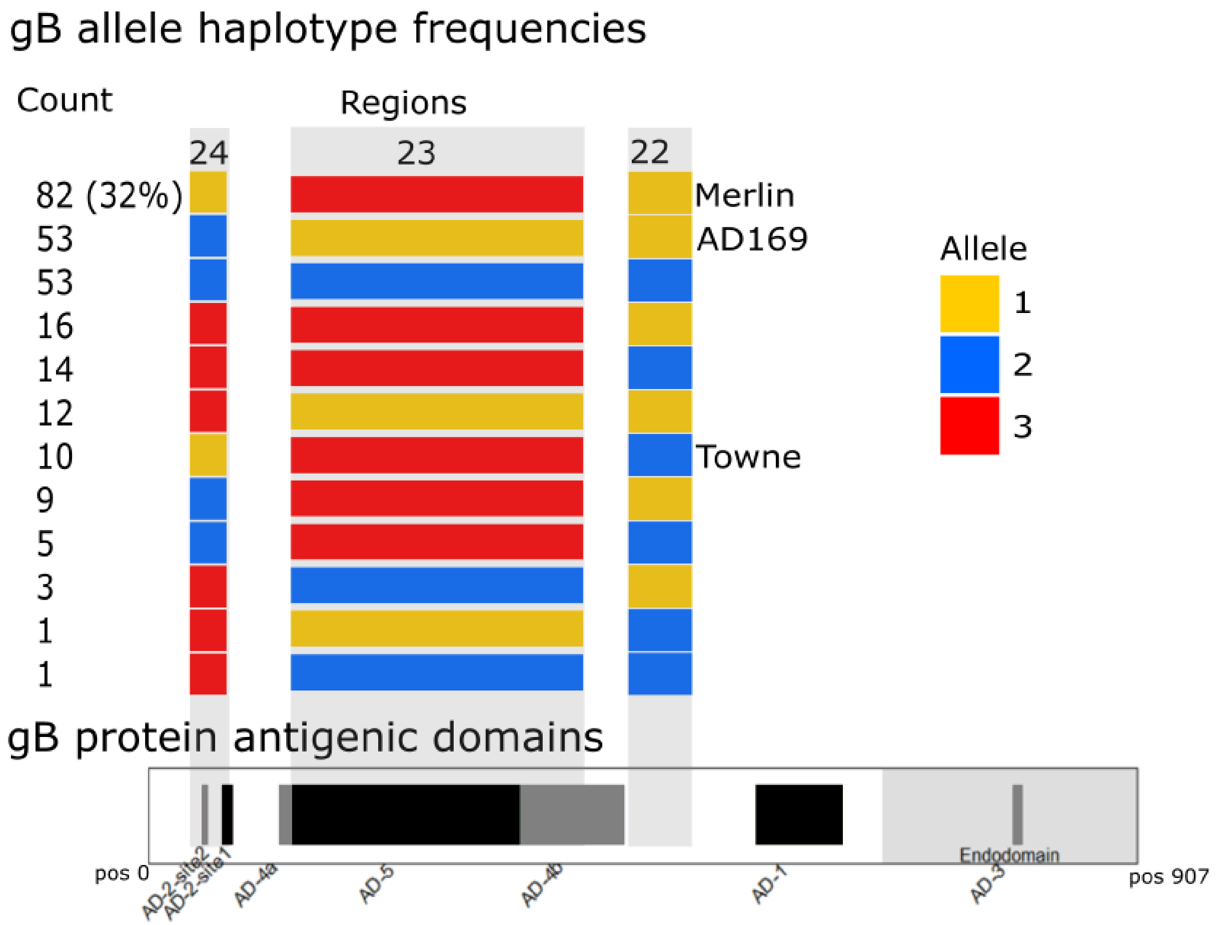

Supplementary Figure 10. Alleles (coloured) in multi-allelic regions 22,23,24 overlap gB Antigenic Domains (AD, greyscale). gB ADs and their corresponding multi-allelic regions are both in frame with the protein residue relative to Towne, light grey long vertical shading emphasises this. The frequency of allele combinations (haplotypes) for the 259 analysed sequences are shown, they are not randomly occurring and reference strains gB haplotypes are also indicated.

Table S1 Variable regions identified by Hidden Markov Modelling

Region	N of Alleles	Start	End	N of Nucleotides	Genes	Chi-squared FDR testing geographic distribution	Allele distribution
1	2	1941	2121	183	RL1	0.356	non-geographic
2	5	5387	6479	1226	**RL5A RL6**	0.009	geographic
3	2	7813	7914	101	RL9A	0.144	non-geographic
4	3	8620	8868	254	RL10	< 0.001	geographic
5	2	9286	9479	193	RL11	0.429	non-geographic
6	5	9840	14133	4760	RL11 **RL12 RL13 UL1** UL2 UL4	1	non-geographic
7	2	14765	14993	232	UL5 UL6	1	non-geographic
8	4	15163	19324	4224	UL10 **UL11** UL6 UL7 UL8 **UL9**	< 0.001	geographic
9	2	24546	24709	163	UL18	1	non-geographic
10	3	25622	26757	1156	UL20	1	non-geographic
11	3	27640	27884	253	UL22A	1	non-geographic
12	**2**	32267	32469	211	UL25	1	non-geographic
13	2	33956	34231	275	UL27	< 0.001	geographic
14	3	43504	44600	1105	UL33	1	non-geographic
15	**2**	48416	48612	212	UL36	0.007	geographic
16	5	50479	51145	682	UL37	1	non-geographic
17	2	53875	54131	257	UL40 UL41A	1	non-geographic
18	2	54736	54916	193	UL41A UL42	1	non-geographic
19	**2**	60433	60720	287	UL45	< 0.001	geographic
20	2	69787	70067	280	UL48	< 0.001	geographic
21	2	71370	71656	294	UL48A	< 0.001	geographic
22	2	82720	83003	283	UL55	1	non-geographic

23	3	83278	84403	1125	UL55	1	non-geographic
24	**3**	84532	84716	202	UL55	< 0.001	geographic
25	2	91851	92082	231		< 0.001	geographic
26	2	93909	94323	423		< 0.001	geographic
27	2	95125	95280	160	RNA4.9	< 0.001	geographic
28	7	107059	109022	1990	**UL74**	< 0.001	geographic
29	2	109129	109426	305	UL75	1	non-geographic
30	2	110100	111111	1011	UL75	1	non-geographic
31	2	111275	111445	173	UL75	1	non-geographic
32	2	112046	112218	172	UL76	< 0.001	geographic
33	2	112991	113433	442	UL77	0.033	geographic
34	2	114235	114372	137	UL78	< 0.001	geographic
35	2	117687	117964	277	UL80 UL80.5	< 0.001	geographic
36	2	119895	120091	196	UL82	< 0.001	geographic
37	2	126001	126092	91	UL86	< 0.001	geographic
38	2	128100	128356	256	UL86	< 0.001	geographic
39	2	128500	128722	222	UL86	< 0.001	geographic
40	2	129087	129283	196	UL86	< 0.001	geographic
41	2	146766	147150	387	UL100	< 0.001	geographic
42	2	166207	166537	354	UL116	1	non-geographic
43	4	168817	170109	1316	UL119 **UL120** UL121	0.05	non-geographic
44	2	171133	171320	187	UL122	0.054	non-geographic
45	2	172835	173287	452	UL122 UL123 UL124	1	non-geographic
46	2	173916	174080	175	UL122 UL123 UL124	< 0.001	geographic
47	2	174252	174465	228	UL124	1	non-geographic
48	2	178839	179201	363	UL132 UL148	1	non-geographic
49	8	180852	181323	726	**UL146** UL147	0.004	geographic

50	3	182416	182725	309	UL144	0.622	non-geographic
51	2	182919	183328	424	UL150A	1	non-geographic
52	3	183904	184172	283	UL142	1	non-geographic
53	4	186573	187057	542	<u>**UL139**</u>	1	non-geographic
54	4	190483	190621	141	UL133	< 0.001	geographic
55	2	190891	191088	198	UL148A UL150A	0.001	geographic
56	2	192291	192441	162	UL150	1	non-geographic
57	3	192991	193241	250	UL150	< 0.001	geographic
58	2	193626	193652	26	UL150	< 0.001	geographic
59	2	194060	195027	2613	UL150	< 0.001	geographic
60	2	197037	197240	302	IRS1	1	non-geographic
61	2	197664	197814	150	IRS1	1	non-geographic
62	2	202772	202925	156	US7	1	non-geographic
63	2	203213	203325	112	US7	1	non-geographic
64	2	208538	208722	184	US14	1	non-geographic
65	2	212019	212119	100	US17	< 0.001	geographic
66	2	223336	223914	579	US26 US27	1	non-geographic
67	3	224108	224224	116	US27	1	non-geographic
68	2	224607	224958	351	US27	1	non-geographic
69	4	225456	225513	57	US27 US28	0.096	non-geographic
70	2	228225	228303	78	US30	1	non-geographic
71	2	230977	231098	124	US33A US34	1	non-geographic
72	4	231565	232587	1527	TRS1	1	non-geographic
73	2	232759	232987	228	TRS1	0.002	Geographic
74	2	233496	233663	253	TRS1	0.058	non-geographic

For each region, the table shows the number of alleles (N of Alleles); the coordinates based on reference sequence NC_006273.2 (Merlin) (start, end); the length of the region (N of Nucleotides); open-reading frames overlapping with the region (Genes). Hypervariable genes previously used by others for CMV genotyping are underlined and

shown in bold. Two additional columns were added to illustrate the geographic allele distribution between Africa and Europe sequences. The Chi-squared FDR: this value represents the result of a Chi-squared test for independence between allele distribution for European and African sequences. To control for multiple comparisons, we used the Benjamini-Hochberg procedure to calculate the false discovery rate (FDR) with a threshold of 0.05. An FDR smaller than 0.05 indicates a significant difference in the allele distributions between the two groups, suggesting a geographically unique allele distribution. Column "Allele distribution" provides a summary of the Chi-squared test. If the FDR value is smaller than 0.05 then the label "geographic" is used; if bigger or equal to 0.05, the label "
non-geographic" is used.

Table S2: The table presents all statistics and parameters from HMMcluster for each multi-allelic region (in addition to Table 1). For each region, the table shows the number of alleles (N of Alleles); the coordinates based on reference sequence NC_006273.2 (Merlin) (start, end); the length of the region (N of Nucleotides); open-reading frames overlapping with the region (Genes).The table also shows for each region the optimal model summarised as: Log of the likelihood that n sequences were derived from m alleles (LLikelihood); The number of parameters required to represent m alleles in a given region (parameters) ; The akaike information criterion of m alleles (AIC); The difference in AIC from 1 allele (AIC_relative)

**Region**	**N of Alleles**	**Start**	**End**	**LLikelihood**	**Parameters**	**AIC**	**AIC_relative**	**N of nucleotides**	**Genes**
1	2	1941	2121	-2244.34	1526	7540.67	-537.04	183	RL1
2	5	5387	6479	-23540.1	27708	102496.3	-123895	1226	RL5A RL6
3	2	7813	7914	-1845.99	828	5347.99	-2098.1	101	RL9A
4	3	8620	8868	-3542.97	3087	13259.94	-2405.78	254	RL10
5	2	9286	9479	-1985.89	1551	7073.78	-638.98	193	RL11
6	5	9840	14133	-124284	104314	457196.2	-492074	4760	RL11 RL12 RL13 UL1 UL2 UL4
7	2	14765	14993	-3221.19	2111	10664.38	-571.01	232	UL5 UL6
8	4	15163	19324	-111383	72346	367457.9	-104602	4224	UL10 UL11 UL6 UL7 UL8 UL9
9	2	24546	24709	-1502.92	1314	5633.85	-1236.82	163	UL18
10	3	25622	26757	-16342.6	14334	61353.15	-31205.3	1156	UL20
11	3	27640	27884	-3053.33	3135	12376.66	-7464.82	253	UL22A
12	2	32267	32469	-2677.94	1762	8879.88	-394.5	211	UL25
13	2	33956	34231	-2356.06	2236	9184.12	-1019.61	275	UL27
14	3	43504	44600	-10912.8	13429	48683.69	-43901.8	1105	UL33
15	2	48416	48612	-3539.6	1766	10611.2	-603.33	212	UL36
16	5	50479	51145	-9250.45	13866	46232.9	-33246.5	682	UL37
17	2	53875	54131	-2258.44	2203	8922.88	-3245.41	257	UL40 UL41A
18	2	54736	54916	-3058.48	1594	9304.95	-585.08	193	UL41A UL42
19	2	60433	60720	-2079.96	2311	8781.92	-1269.63	287	UL45
20	2	69787	70067	-1316.08	2253	7138.15	-297.48	280	UL48
21	2	71370	71656	-3531.28	2396	11854.57	-589.18	294	UL48A
22	2	82720	83003	-1711.08	2283	7988.16	-840.2	283	UL55
23	3	83278	84403	-8685.43	13608	44586.85	-22415.4	1125	UL55
24	3	84532	84716	-2648.63	2522	10341.27	-7573.54	202	UL55

25	2	91851	92082	-1590.76	1860	6901.53	-1338.33	231	
26	2	93909	94323	-7853.7	3584	22875.4	-1354.6	423	
27	2	95125	95280	-1888.98	1316	6409.96	-730.67	160	RNA4.9
28	7	107059	109022	-26242.7	56877	166239.4	-101446	1990	UL74
29	2	109129	109426	-2611.98	2475	10173.96	-479.55	305	UL75
30	2	110100	111111	-5666.96	8243	27819.92	-11452.5	1011	UL75
31	2	111275	111445	-1794.94	1404	6397.88	-4866.71	173	UL75
32	2	112046	112218	-1647.97	1381	6057.94	-1982.39	172	UL76
33	2	112991	113433	-3350.31	3550	13800.62	-245.41	442	UL77
34	2	114235	114372	-1198.08	1100	4596.15	-1620.46	137	UL78
35	2	117687	117964	-1386.25	2225	7222.51	-503.99	277	UL80 UL80.5
36	2	119895	120091	-1409.05	1582	5982.11	-342.84	196	UL82
37	2	126001	126092	-563.04	734	2594.07	-2121.15	91	UL86
38	2	128100	128356	-901.27	2054	5910.54	-496.53	256	UL86
39	2	128500	128722	-1277.87	1791	6137.73	-522.06	222	UL86
40	2	129087	129283	-665.98	1580	4491.95	-585.33	196	UL86
41	2	146766	147150	-2115.24	3117	10464.47	-3332.13	387	UL100
42	2	166207	166537	-4356.6	2954	14621.2	-3202.23	354	UL116
43	4	168817	170109	-19312.7	21443	81511.3	-33167.9	1316	UL119 UL120 UL121
44	2	171133	171320	-1307.13	1499	5612.25	-855.49	187	UL122
45	2	172835	173287	-4440.12	3646	16172.23	-2648.31	452	UL122 UL123 UL124
46	2	173916	174080	-2616.39	1454	8140.77	-1872.19	175	UL122 UL123 UL124
47	2	174252	174465	-1741.2	1871	7224.4	-961.63	228	UL124
48	2	178839	179201	-5352.81	2931	16567.62	-3125.15	363	UL132 UL148
49	8	180852	181323	-21661.8	26161	95645.63	-61020.5	726	UL146 UL147
50	3	182416	182725	-3995.92	3864	15719.83	-29319.6	309	UL144
51	2	182919	183328	-8276.39	3989	24530.79	-5389.35	424	UL150A
52	3	183904	184172	-3960.88	3706	15333.76	-12722.1	283	UL142

53	4	186573	187057	-10894.7	9545	40879.45	-40120.7	542	UL139
54	4	190483	190621	-3982.02	2328	12620.05	-4712.79	141	UL133
55	2	190891	191088	-3392.1	1623	10030.2	-645.52	198	UL148A UL150A
56	2	192291	192441	-3855.16	1352	10414.33	-920.54	162	UL150
57	3	192991	193241	-4196.98	3046	14485.95	-2851.04	250	UL150
58	2	193626	193652	-589.88	210	1599.76	-206.58	26	UL150
59	2	194060	195027	-92540.9	25697	236475.8	-3343.18	2613	UL150
60	2	197037	197240	-7399.69	2758	20315.38	-691.99	302	IRS1
61	2	197664	197814	-1286.64	1204	4981.28	-1395.98	150	IRS1
62	2	202772	202925	-1946.16	1425	6742.32	-1488.1	156	US7
63	2	203213	203325	-1589.86	956	5091.71	-483.36	112	US7
64	2	208538	208722	-1420.51	1477	5795.02	-1062.52	184	US14
65	2	212019	212119	-578.16	810	2776.32	-1716.71	100	US17
66	2	223336	223914	-3050.43	4650	15400.85	-1772.27	579	US26 US27
67	3	224108	224224	-1893.07	1506	6798.15	-6150.59	116	US27
68	2	224607	224958	-2168.77	2819	9975.54	-5078.58	351	US27
69	4	225456	225513	-524.96	916	2881.92	-2445.6	57	US27 US28
70	2	228225	228303	-1052.07	637	3378.13	-465.97	78	US30
71	2	230977	231098	-1501.77	1012	5027.55	-1420.82	124	US33A US34
72	4	231565	232587	-46165.9	28262	148855.8	-50024.5	1527	TRS1
73	2	232759	232987	-1967.37	1826	7586.73	-447.88	228	TRS1
74	2	233496	233663	-6240.4	2382	17244.8	-973.35	253	TRS1
